# Diagnostic Performance of Deep Learning and Radiomics in Extracranial Carotid Plaque Detection: Systematic Review and Meta-Analysis

**DOI:** 10.2196/77092

**Published:** 2026-01-22

**Authors:** Lingjie Ju, Yongsheng Guo, Haiyong Guo, Ruijuan Liu, Yiyang Wang, Siyu Wang, Na Ma, Junhong Ren

**Affiliations:** 1 Department of Sonography, Beijing Hospital, National Center of Gerontology, Institute of Geriatric Medicine, Chinese Academy of Medical Sciences & Peking Union Medical College, 1 Dahua Road, Dongdan, Dongcheng District, Beijing, 100730, China, 86 13910813603; 2 Department of Sonography, Beijing Hospital, National Center of Gerontology, Institute of Geriatric Medicine, Peking University Health Science Center, Beijing, China; 3 Department of Sonography, Beijing Hospital, National Center of Gerontology, Institute of Geriatric Medicine, Chinese Academy of Medical Sciences, Beijing, China

**Keywords:** extracranial carotid plaque, deep learning, radiomics, systematic review, diagnosis

## Abstract

**Background:**

Artificial intelligence–enhanced imaging techniques have demonstrated promising diagnostic potential for carotid plaques, a key cardiovascular and cerebrovascular risk factor. However, previous studies did not systematically synthesize their diagnostic accuracy.

**Objective:**

This study aimed to quantitatively explore the diagnostic efficacy of deep learning (DL) and radiomics for extracranial carotid plaques and establish a standardized framework for improving plaque detection.

**Methods:**

We searched the PubMed, Embase, Cochrane, Web of Science, and Institute of Electrical and Electronics Engineers databases to identify studies involving the use of radiomics or DL models to diagnose extracranial carotid artery plaques from inception up to September 24, 2025. The quality of the studies was determined using Quality Assessment of Diagnostic Accuracy Studies for Artificial Intelligence (QUADAS-AI). A meta-analysis was conducted using StataMP (version 17.0; StataCorp) with a bivariate mixed-effects model to calculate pooled sensitivity and specificity, generate summary receiver operating characteristic (SROC) curves, assess Cochran *Q* statistic and *I*²-based heterogeneity, and conduct subgroup analyses and regression analysis.

**Results:**

Among 40 studies comprising 17,246 patients, 34 integrated independent test sets or validation sets in the quantitative statistical analysis. Among them, 24 focused on DL models, 10 on machine learning models based on radiomics. The combined sensitivity, specificity, and area under the SROC curve were 0.88 (95% CI 0.85‐0.91; *P*<.001; *I*^2^=93.58%), 0.89 (95% CI 0.85‐0.92; *P*<.001; *I*^2^=91.38%), and 0.95 (95% CI 0.92‐0.96), respectively. Compared with the machine learning models based on radiomics algorithms, DL models achieved comparable improvements in specificity and area under the SROC curve. It was observed that transfer learning and a large sample size enhanced the diagnostic performance of models. Models used to identify plaque stability and presence had similar diagnostic performances, both of which were more effective in identifying symptomatic plaque models. A total of 7 studies demonstrated that the models that combined clinical features exhibited comparable diagnostic capability to pure DL and radiomics models. Additionally, 7 studies performed external validation, obtaining lower diagnostic performance than in testing groups. Limited regression analysis failed to identify significant sources of heterogeneity, and the limited number of eligible studies restricted more comprehensive subgroup analyses. The high heterogeneity in the study results may be due to different scanning parameters, model architecture, image segmentation, and algorithms.

**Conclusions:**

Radiomics algorithms and DL models can effectively diagnose extracranial carotid plaque. However, there are concerns regarding irregularities in research design and the absence of multicenter studies and external validation. Future research should aim to reduce bias risk and enhance the generalizability and clinical orientation of the models.

## Introduction

Extracranial carotid plaques are biomarkers of coronary artery disease and cerebral ischemic events, including ischemic heart disease and stroke. The global prevalence of carotid plaques among individuals aged 30‐79 years is estimated at 21.1% (n=815.76 million) in 2020. This high prevalence reflects a growing global burden of cardiovascular and cerebrovascular diseases, posing a significant challenge to public health systems [1]. Therefore, early detection and management of carotid plaque can potentially reduce the risk of stroke and cardiovascular events [2-4], and thus, effective detection and classification technologies need to be prioritized.

Imaging methods for carotid plaque imaging, such as ultrasound, computed tomography angiography (CTA), magnetic resonance imaging (MRI), and digital subtraction angiography, facilitate detection, stenosis assessment, and plaque composition analysis [5]. Conventional ultrasound is the first-line screening method [6]. Studies show that periapical radiographs (PRs) can serve as a supplementary screening tool, demonstrating a 50% concordance with ultrasound or CTA [7-9]. Current imaging primarily identifies high-risk features, such as plaque neovascularity, lipid-rich necrotic cores, thin fibrous caps, and intraplaque hemorrhage plaque ulceration [4,10]. Among them, the contrast-enhanced ultrasound or superb microvascular imaging can accurately quantify neovascularization and correlates well with histopathology [11-14], offering rapid, noninvasive, and reliable quantification [15]. It is proficient in vascular imaging and ulcer detection [16], as well as stenosis assessment [17], but it faces challenges with small lipid cores and thin fibrous caps [18]. MRI remains the gold standard for assessing plaque composition, particularly for identifying lipid cores and intraplaque hemorrhage [19]. While digital subtraction angiography is the reference standard, its invasive nature limits its application. Notably, the accuracy of these diagnostic techniques largely relies on the expertise of imaging or clinical physicians, which causes inconsistencies in the assessment results of carotid atherosclerotic plaques—particularly in measuring carotid intima-media thickness, characterizing intraplaque components, and evaluating fibrous cap integrity.

The radiomics algorithms and deep learning (DL) models have demonstrated significant potential in medical image analysis [20]. Radiomics is a quantitative medical imaging analysis approach that aims to transform high-dimensional image features (such as texture heterogeneity, spatial topological relationships, and intensity distribution) into quantifiable digital biomarkers, thereby providing objective evidence to guide clinical decision-making. However, the characteristic dimensionality of radiomics data often far exceeds sample sizes, which renders the traditional statistical methods inadequate [21]. Machine learning (ML), with the potential to process large-scale, high-dimensional data and uncover deep correlations among these complex features [22]. Combining radiomics with ML to develop an ML model using radiomics can enhance the diagnostic performance of AI in large and complex datasets, exceeding the performance of models constructed through traditional statistical methods.

DL is also one of the important subbranches of artificial intelligence, which can automatically learn and layer from raw data without manual design of features, ultimately generating predictions via an output layer [23]. DL-driven image generation techniques have demonstrated remarkable effectiveness in cross-modality imaging and synthesis tasks across various sequences within the same modality. With the rapid development of computer technology, ML models based on radiomics and DL models based on radiomics have become important tools for cardiovascular disease research. Current evidence suggests that these methods can significantly improve the quantitative assessment accuracy of atherosclerotic plaque progression and enhance the diagnostic and predictive power of major adverse cardiovascular events [24-26]. In recent years, research on the application of these methods in the fields of plaque diagnosis, stability assessment, and symptomatic plaque identification has increased significantly. Although these advancements have significantly improved the diagnosis of carotid plaques, variations in data dependency and imaging configurations among different models create inconsistencies in diagnostic accuracy. Moreover, these models may become overly specialized in common imaging configurations, even when using radiomics data from identical sources. Currently, systematic evaluations of its clinical validity remain limited.

Therefore, this systematic review comprehensively assesses the applications of ML models based on radiomics algorithms and DL models in carotid plaques, while highlighting gray areas in the available literature.

## Methods

### Study Registration

The study was performed in line with the PRISMA-DTA (Preferred Reporting Items for Systematic Reviews and Meta-Analyses of Diagnostic Test Accuracy Studies) guidelines [27] and PRISMA (Preferred Reporting Items for Systematic Reviews and Meta-Analyses) standards [28,29] and was registered on the International Prospective Register of Systematic Reviews (PROSPERO CRD42025638492).

### Data Sources and Search Strategy

Relevant articles were searched on PubMed, Embase, Web of Science, Cochrane Library, and Institute of Electrical and Electronics Engineers (IEEE) databases, focusing on English-language articles published up to September 24, 2025. The literature search was based on the PIO (population, intervention, and outcomes) principles: “P” represents carotid artery disease, carotid plaques, or atherosclerosis populations; “I” represents radiomics or DL as interventions; and “O” represents the outcomes of diagnosis and their subordinates and other keywords. Furthermore, we manually analyzed the reference lists of all included articles to identify additional relevant publications. The complete search strategy is outlined in Table S1 in Multimedia Appendix 1. The EndNote 20 software (Clarivate Analytics) was used to manage the included studies.

### Eligibility Criteria

#### Inclusion Criteria

The inclusion criteria included:

1. Studies on patients with extracranial carotid plaques that aimed to detect or distinguish between unstable and symptomatic plaques, among other factors.
2. Studies using radiomics algorithms or DL models based on medical imaging techniques, such as ultrasound, CTA, or MRI, to diagnose carotid plaques.
3. Studies reported the diagnostic performance metrics, including confusion matrix, 2×2 diagnostic tables, accuracy, sensitivity, specificity, receiver operating characteristic (ROC) curves, *F*_1_-score, precision, recall, etc.
4. Those that adopted the following designs: prospective or retrospective cohorts, diagnostic accuracy trials, model development or validation studies, and comparative studies (eg, AI models vs AI models combined with clinical features).
5. Only studies published in English and with extractable quantitative data were deemed eligible.

#### Exclusion Criteria

The exclusion criteria excluded:

1. Studies involving nonhuman subjects (animal experiments or in vitro models), those that explored intracranial or coronary plaques, enrolled pediatric populations (<18 years), or reported only generalized atherosclerosis without plaque-specific criteria (focal intima-media thickness ≥1.5 mm) or specific diagnostic metrics;
2. Those that did not adopt well-defined deep learning models or radiomics algorithms, focused only on image segmentation or texture analysis without diagnostic validation, or reported predictive models without providing a clear diagnostic relevance.
3. Studies that lacked a validated reference standard.
4. Studies that did not report diagnostic performance.
5. Informal publication types (eg, reviews, letters to the editor, editorials, and conference abstracts).
6. Studies that did not report validation or test sets.

### Screening of Articles and Data Extraction

In the initial screening, duplicates were excluded followed by reading of full texts, and data were entered into a predefined extraction table, which included surnames of authors, source of data, publication year, algorithm architecture, type of internal validation, availability of open access data, external verification status, reference standard, transfer learning application, number of cases for training, test, internal, or external validation, study design, sample size, mean or median age, inclusion criteria, and model evaluation metrics. The contingency tables are derived from the models explicitly identified by the original authors as the best-performing ones. Data from external validation sets were prioritized. If there were no external validation set in the original studies, data from internal validation sets were used. If neither was available, the contingency tables corresponding to the test sets were selected. This process was performed by two researchers (LJ and YG), working independently, and any differences were resolved through discussion with a third researcher (HG).

### Quality Assessment

Two blinded investigators (LJ and YG) systematically assessed the quality of studies using the Quality Assessment of Diagnostic Accuracy Studies for Artificial Intelligence (QUADAS-AI) tool. Specifically, they evaluated the risk of bias and applicability concerns across 4 domains: flow and timing, reference standard, index test, and participant selection. Although the Quality Assessment of Diagnostic Accuracy Studies-2 (QUADAS-2) is extensively applied to assess the quality of diagnostic accuracy studies [30], it does not address the specific methodological choices, result analyses, and measurements related to diagnostic studies using AI. To address this gap, QUADAS-AI was developed as a consensus-based tool to aid readers in systematically examining the risk of bias and the usability of AI-related diagnostic accuracy studies (Table S6 in Multimedia Appendix 1) [31], thereby improving the quality assessment process [32,33]. Any evaluation discrepancies were resolved by a third investigator (HG).

### Statistical Analysis

A meta-analysis was performed using STATA/MP software (version 17.0; Stata Corporation) with a bivariate random-effects model. For meta-analyses of the diagnostic accuracy of AI-based models, bivariate mixed-effects models can account for both within-study variability (random effects) and between-study heterogeneity (fixed effects), ensuring the robustness of the pooled estimates [34]. A contingency table was generated using data from the included literature, and then we calculated metrics such as the number of cases, the Youden index, sensitivity, specificity, and recall. The diagnostic efficacy of radiomics algorithms and DL models in evaluating carotid plaque was determined using a summary receiver operating characteristic (SROC) curve and area under the curve (AUC; 0.7≤AUC<0.8 fair; 0.8≤AUC<0.9 good; and AUC≥0.9 excellent). Publication bias was explored using Deeks funnel plot asymmetry test. The Fagan nomogram was developed to determine clinically pertinent posttest probabilities (P-post) and likelihood ratios (LRs). LRs were determined by comparing the probability of test results between diseased and nondiseased groups. The pretest probability was subsequently adjusted based on test results and LRs to obtain P-post [35]. The Cochran *Q* (*P*≤.05) and *I*^2^ statistic were used to explore heterogeneity among the included studies, and regression analysis was conducted to assess sources of heterogeneity. *I*^2^≤50% indicated mild heterogeneity, 50%<*I*^2^<75% reflected moderate heterogeneity, and *I*^2^≥75% indicated high heterogeneity.

The subgroup analysis encompassed the following factors: (1) model type (DL or ML model), (2) medical imaging modalities (PRs, ultrasound, MRI, or CTA), (3) application of transfer learning, (4) characteristics of carotid plaques (presence vs absence, stable vs vulnerable, and symptomatic vs asymptomatic), (5) comparison of the most effective ML model based on radiomics algorithm and DL models using the same dataset and clinicians’ diagnoses, (6) different types of datasets (testing and validation), (7) low and high or unclear risk of bias studies, (8) different sample sizes of model, and (9) models with different research designs (multicenter studies and single-center studies). To identify the sources of heterogeneity associated with nonthreshold effects, meta-regression was performed using the above-mentioned covariates.

Sensitivity analysis was performed to assess the stability of the results by several steps: (1) excluding specific articles one by one to determine the stability of the results, (2) excluding studies with extremely large sample sizes (N≥500; n=7 studies), (3) excluding studies with extremely small sample sizes (N≤50; n=4 studies), and (4) excluding studies with extreme effect sizes (sensitivity or specificity>0.95 or <0.7; n=11 studies).

## Results

### Study Selection

**Figure 1.**
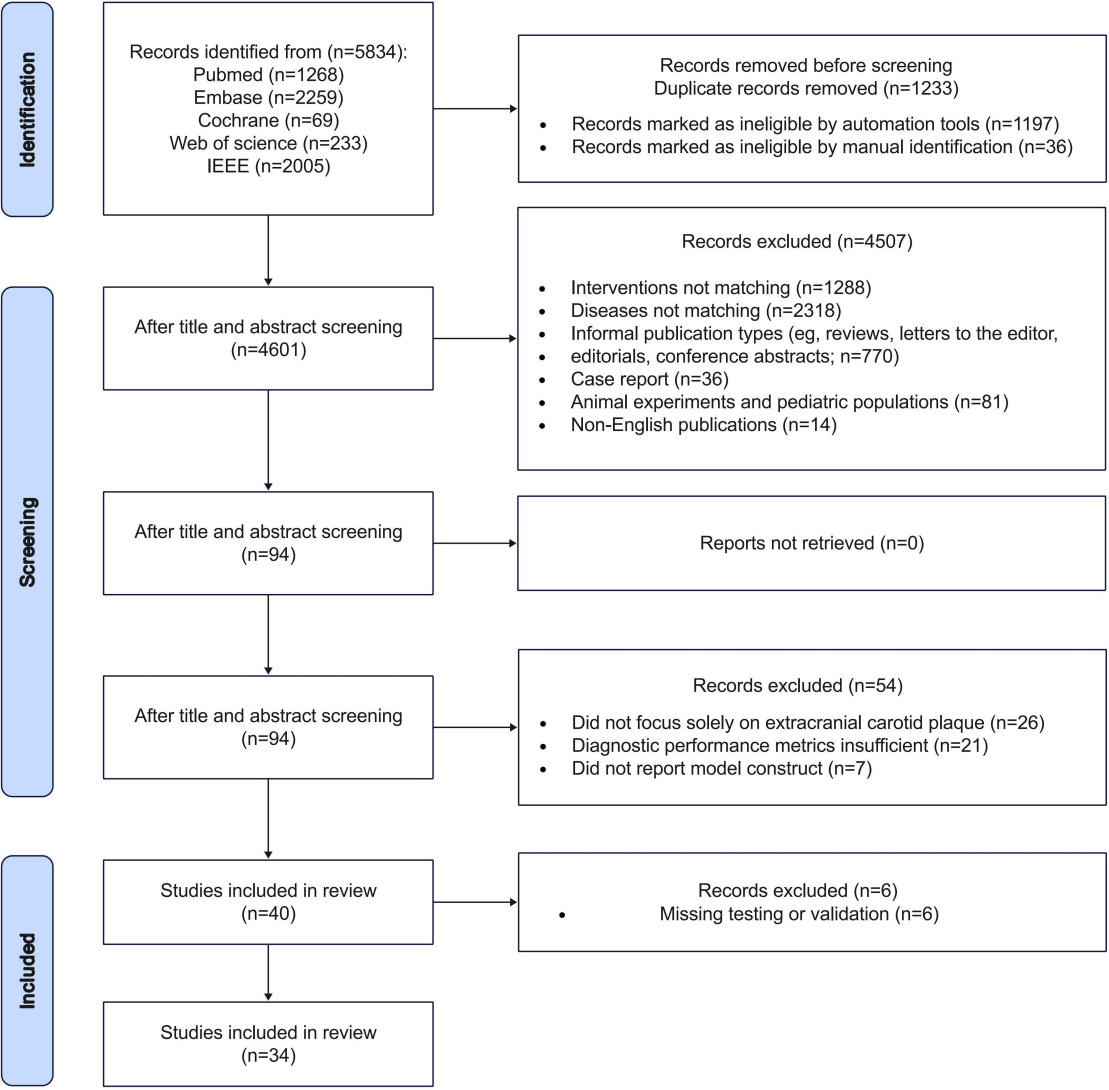
PRISMA (Preferred Reporting Items for Systematic Reviews and Meta-Analyses) flowchart of study selection. IEEE: Institute of Electrical and Electronics Engineers.

We obtained 5834 studies in the initial analysis, of which 1233 were excluded for duplication or redundancy. After screening titles and abstracts, 4507 publications were eliminated. After the full texts of the 94 articles were read, 40 studies were eligible for meta-analysis. The PRISMA flow diagram of the study showing the selection process is presented in Figure 1.

### Study Characteristics

Among the 40 studies that fulfilled the systematic review’s inclusion criteria, 34 provided sufficient quantitative data (contingency tables from validation or test sets) eligible for incorporation into the meta-analysis. The detailed characteristics of all 40 eligible studies are summarized in Tables S3 and S4 in Multimedia Appendix 1, while all subsequent quantitative analyses were conducted based on the 34 studies with available quantitative data. Overall, 34 studies were included [36-69], among which 9 were multicenter studies [41,43,45,49,57,63-65,69], 3 used public databases [37,40,53], 13 provided open access to the data [37,40,45,48-50,53,57,59,63-66]. A total of 12 studies conducted internal validation [38,39,41,42,44,47,48,57,61,64,69,70] to confirm the reproducibility of the model development process and prevent overfitting. In addition, 7 studies conducted external validation [41,50,57,60,63,64,69] to assess the model’s transportability and generalizability using unused datasets. Only 1 study conducted a comparative analysis of the diagnostic performance of DL models with that of clinicians [57]. The medical imaging modalities included PRs (n=5), ultrasound (n=16), MRI (n=5), and CTA (n=8). The core features of the 34 studies are presented in Tables1  2, with further details provided in Tables S2 and S3 in Multimedia Appendix 1.

**Table 1.** Data sources, indicators, and algorithms of included studies.

Study, year	Data source				Validation type
	Source of data	Number of cases for training, test, internal, or external	Data range	Labels	
Su et al [51], 2023	China	322; 138; NR^a^; NR	NR	Stable or vulnerable plaque	No
Zhang et al [70], 2024	China	4064; NR; 1016; NR	NR	Stable or vulnerable plaque	Internal validation
Zhou et al [44], 2024	China	751; 261; 258; NR	NR	Stable or vulnerable plaque	Internal validation
Zhang et al [58], 2021	China	121; 41; NR; NR	NR	Symptomatic or asymptomatic	No
Zhai et al [41], 2024	NR	240; NR; 60; 100	January 2017-January 2022	Normal or abnormal	External validation
Yoo et al [39], 2024	South Korea	388; 130; 130; NR	2009‐2022	Normal or abnormal	Internal validation
Xu et al [56], 2022	NR	NR	NR	Stable or vulnerable plaque	No
Xie et al [47], 2023	China	264; 75; 38; NR	2020‐2021	Stable or vulnerable plaque	Internal validation
Wei et al [62], 2024	China	2725; 554; NR; NR	NR	Normal or abnormal	No
Ganitidis et al [60], 2021	Greece	46; 10; 18; NR	NR	Symptomatic or asymptomatic	Internal validation
Shi et al [50], 2023	China	134; 33; NR; NR	October 2019-July 2022	Symptomatic or asymptomatic	No
Gui et al [49], 2023	China	84; 20; NR; NR	NR	Symptomatic or asymptomatic	No
Ali et al [71], 2024	Italy	336; 84; NR; NR	NR	Symptomatic or asymptomatic	No
Amitay et al [48], 2023	Israel	371; 144; 144; NR	2016‐2021	Normal or abnormal	Internal validation
Ayoub et al [72], 2023	China	136; 150; 69; NR	NR	Stable or vulnerable plaque	Internal validation
Cilla et al [55], 2022	Italy	NR	October 2015-October 2019	Stable or vulnerable plaque	No
Guang et al [57], 2021	China	136; NR; 69; NR	September 2017-September 2018	Stable or vulnerable plaque	Internal validation
He et al [69], 2024	China	3088; NR; 772; 1564	January 2021-March 2023	Normal or abnormal; stable or vulnerable plaque	Internal and external validation
Latha et al [73], 2021	India	NR	NR	Normal or abnormal	No
Ma et al [59], 2021	China	1169; 294; NR; NR	NR	A total of 3 types (echo-rich, intermediate, and echolucent)	No
Pisu et al [43], 2024	Italy	163; 106; NR; NR	March 2013-October 2019	Symptomatic or asymptomatic	No
Wang et al [74], 2024	China	154; 39; NR; NR	January 1, 2018-December 31, 2021	Symptomatic or asymptomatic	No
Gago et al [53], 2022	Spain	NR	2007‐2010	Normal or abnormal	No
Omarov et al [40], 2024	The United Kingdom	577; 103; NR; NR	NR	Normal or abnormal	No
Wang et al [45], 2023	China	2619; 1122; NR; NR	NR	Stable or vulnerable plaque	No
Vinayahalingam et al [42], 2024	Germany	280; 37; 37; NR	NR	Normal or abnormal	No
Singh et al [37], 2024	Cyprus; The United Kingdom; NR	3088; 772; NR; NR	NR	Stable or vulnerable plaque	No
Shan et al [46], 2023	China	52; 22; NR; NR	January 2018-December 2021	Stable or vulnerable plaque	No
Li et al [38], 2024	NR	4546; 1471; 1019; NR	NR	Normal or abnormal	Internal validation
Jain et al [54], 2021	NR	682; 76; NR; NR	July 2009-September 2010	Stable or vulnerable plaque	No
Molinari et al [36], 2018	Italy	NR	2004‐2010	Symptomatic or asymptomatic	No
Kats et al [61], 2019	Israel	1946; 7; 12; NR	NR	Normal or abnormal	Internal validation
Chen et al [52], 2022	China	81; 34; NR; NR	July 2015-May 2021	Symptomatic or asymptomatic	No
Zhao et al [63], 2025	China	317; NR; NR; 328	January 2018-December 2023 (Center 1); Jan 2022-December 2023 (Center 2,3)	Symptomatic or asymptomatic	External validation
Hu et al [64], 2025	China	213; NR; 93; 110	January 2018-May 2023 (Center 1); January 2020-May 2023 (Center 2)	Symptomatic or asymptomatic	Internal and external validation
Li et al [75], 2025	China	2069; 887; NR; NR	October 2021-January 2022	normal or abnormal	No
Yu et al [66], 2025	China	146; 63; NR; NR	April 2022-August 2023	HIPs^b^ or NHIPs^c^	No
Liapi et al [65], 2025	Cyprus, The United Kingdom, and Greece	168; 46; 22; NR	NR	Symptomatic or asymptomatic	Internal validation
Kuwada et al [67], 2025	Japan	Training and validation data: 500; Test data: 80	2008‐2023	Normal or abnormal	No
Lao et al [68], 2025	China	76; 31; NR; NR	January 2017-October 2022	Stable or vulnerable plaque	No

a NR: not reported.

b HIP: highly inflammatory plaque.

c NHIP: non–highly inflammatory plaque.

**Table 2.** Data sources, indicators, and algorithms of all studies.

Study, year	Indicator definition		Algorithm		
	Device	Exclusion of poor quality cases	Algorithm architecture	ML^a^ or DL^b^	Transfer learning applied
Su et al [51], 2023	Ultrasound	NR^c^	Inception V3; VGG-16^d^	DL	No
Zhang et al [70], 2024	Ultrasound	NR	Fusion-SSL	DL	No
Zhou et al [44], 2024	Ultrasound	NR	Tri-Correcting	DL	No
Zhang et al [58], 2021	MRI^e^	Yes	LASSO^f^ MRI-based model (HRPMM^g^)	ML models based on radiomics algorithms^h^ (LASSO algorithm)	No
Zhai et al [41], 2024	CT	Yes	3D-UNet; ResUNet	DL	No
Yoo et al [39], 2024	PRs	Yes	CACSNet	DL	Yes
Xu et al [56], 2022	Ultrasound	NR	Multi-feature fusion method	DL	No
Xie et al [47], 2023	Ultrasound	NR	CPTV^i^	DL	No
Wei et al [62], 2024	Ultrasound	Yes	BETU^j^	DL	Yes
Ganitidis et al [60], 2021	Ultrasound	NR	CNNs^k^	DL	No
Shi et al [50], 2023	CT^l^ and MRI	Yes	LASSO regression	ML models based on radiomics algorithms (LASSO algorithm)	No
Gui et al [49], 2023	MRI	Yes	3D-SE-DenseNet121^m^; ANOVA_spearman_LASSO and MLP^n^	ML models based on radiomics algorithms (LASSO, ANOVA_LASSO and ANOVA_spearman_LASSO) and DL	No
Ali et al [71], 2024	Ultrasound	No	CAROTIDNet^o^	DL	No
Amitay et al [48], 2023	PRs	Yes	InceptionResNetV2 (minimum-maximum)	DL	Yes
Ayoub et al [72], 2023	MRI	NR	HViT^p^	DL	No
Cilla et al [55], 2022	CT	Yes	SVM RBF^q^ kernel	ML models based radiomics algorithms (logistic regression [LR]), support vector machine (SVM), and CART^r^	No
Guang et al [57], 2021	Ultrasound	Yes	DL-DCCP^s^	DL	Yes
He et al [69], 2024	Ultrasound	Yes	BCNN^t^-ResNet^u^	DL	No
Latha et al [73], 2021	Ultrasound	NR	CART; logistic regression; random forest; CNN; Mobilenet; Capsulenet	ML models based radiomics algorithms (CART, logistic regression, and random forest algorithm) and DL	Yes
Ma et al [59], 2021	Ultrasound	NR	MSP^v^-VGG	DL	Yes
Pisu et al [43], 2024	CT	Yes	GB-GAM^w^	ML models based radiomics algorithms (NR)	No
Wang et al [74], 2024	CT	Yes	SR^x^	DL	Yes
Gago et al [53], 2022	Ultrasound	NR	End-to-end framework	DL	No
Omarov et al [40], 2024	Ultrasound	Yes	YOLOv8^y^	DL	Yes
Wang et al [45], 2023	MRI	Yes	ResNet-50	DL	Yes
Vinayahalingam et al [42], 2024	PRs^z^	Yes	Faster R-CNN^aa^ with Swin Transformer (Swin-T)	DL	Yes
Singh et al [37], 2024	Ultrasound	Yes	GoogLeNet^ab^	ML models based on radiomics algorithms (SVM algorithms) and DL	Yes
Shan et al [46], 2023	CT and ultrasound	Yes	LR^ac^; SVM^ad^; RF^ae^; LGBM^af^; daBoost; XGBoost^ag^; MLP	ML models based on radiomics algorithms (Pyradiomics package in Python software)	Yes
Li et al [38], 2024	Ultrasound	NR	U-Net; CNN	DL	No
Jain et al [54], 2022	Ultrasound	NR	SegNet-UNet^ah^	DL	No
Molinari et al [36], 2018	Ultrasound	NR	SVM	ML models based on radiomics algorithms (BEMD^ai^)	No
Kats et al [61], 2019	PRs	NR	Faster R-CNN	DL	No
Chen et al [52], 2022	MRI	Yes	LASSO	ML models based on radiomics algorithms (mRMR^aj^ algorithm and LASSO algorithm)	No
Zhao et al [63], 2025	CTA^ak^	Yes	XGBoost	ML models based on radiomics algorithms (XGBoost)	No
Hu et al [64], 2025	CTA	Yes	LASSO regression; SVM; logistic regression	ML models based on radiomics algorithms (LASSO algorithm) and classifier (SVM)	No
Li et al [75], 2025	Ultrasound	NR	XGBoost; RF; LASSO regression	ML models based on radiomics algorithms (XGBoost, RF, LASSO regression)	No
Yu et al [66], 2025	MRI	Yes	Plaque-R model; PVAT-R^al^ model; ensemble model	ML models based on radiomics algorithms (LASSO algorithm) and ensemble learning	No
Liapi et al [65], 2025	Ultrasound	NR	Xception	DL	Yes
Kuwada et al [67], 2025	Ultrasound	NR	GoogLeNet; YOLOv7	DL	No
Lao et al [68], 2025	CTA	Yes	mRMR algorithm; LASSO regression	ML models based on radiomics algorithms (mRMR algorithm; LASSO algorithm)	No

a ML: machine learning.

b DL: deep learning.

c NR: not reported.

d VCG: VGG visual geometry group network.

e MRI: magnetic resonance imaging.

f LASSO: least absolute shrinkage and selection operator.

g HRPMM: high-risk plaque MRI-based model.

h Definition of ML models based on radiomics algorithms and deep learning (DL): ML models based on radiomics algorithms are models that rely on artificially designed features (such as texture and shape features) and use traditional algorithms (such as random forest, support vector machine, logistic regression, etc) to complete classification, without the need for DL algorithms to be in the core task. The DL model was defined as a model that automatically extracts features and completes classification through neural networks (such as convolutional neural network, ResNet, etc), regardless of whether the input contains a small number of artificial features, as long as the core task relies on the DL algorithm.

i CPTV: classification of plaque by tracking videos.

j BETU: be easy to use.

k CNN: convolutional neural network.

l CT: computed tomography.

m 3D-SE-DenseNet121: 3D squeeze-and-excitation DenseNet with 121 layers.

n MLP: multilayer perceptron.

o CAROTIDNet: carotid symptomatic/asymptomatic plaque detection network.

p HViT: hybrid vision transformer.

q SVM RBF: kernel support vector machine with radial basis function kernel.

r CART: classification and regression tree.

s DL-DCCP: deep learning-based detection and classification of carotid plaque.

t BCNN: bilinear convolutional neural network.

u ResNet: deep residual network.

v MSP: multilevel strip pooling.

w GB-GAM: gradient-boosting generalized additive model.

x SR: super resolution.

y YOLOv8: you only look once version 8.

z PR: panoramic radiograph.

aa Faster R-CNN: faster region-based convolutional network.

ab GoogLeNet: Google network.

ac LR: logistic regression.

ad SVM: support vector machine.

ae RF: random forest.

af LGBM: light gradient boosting machine.

ag XGBoost: extreme gradient boosting.

ah SegNet-UNet: segmentation network-UNet.

ai BEMD: bidimensional empirical mode decomposition.

aj mRMR: minimum redundancy maximum relevance.

ak CTA: computed tomography angiography.

al PVAT: perivascular adipose tissue.

### Meta-Analysis of Diagnostic Performance

#### Synthesized Results

**Figure 2.**
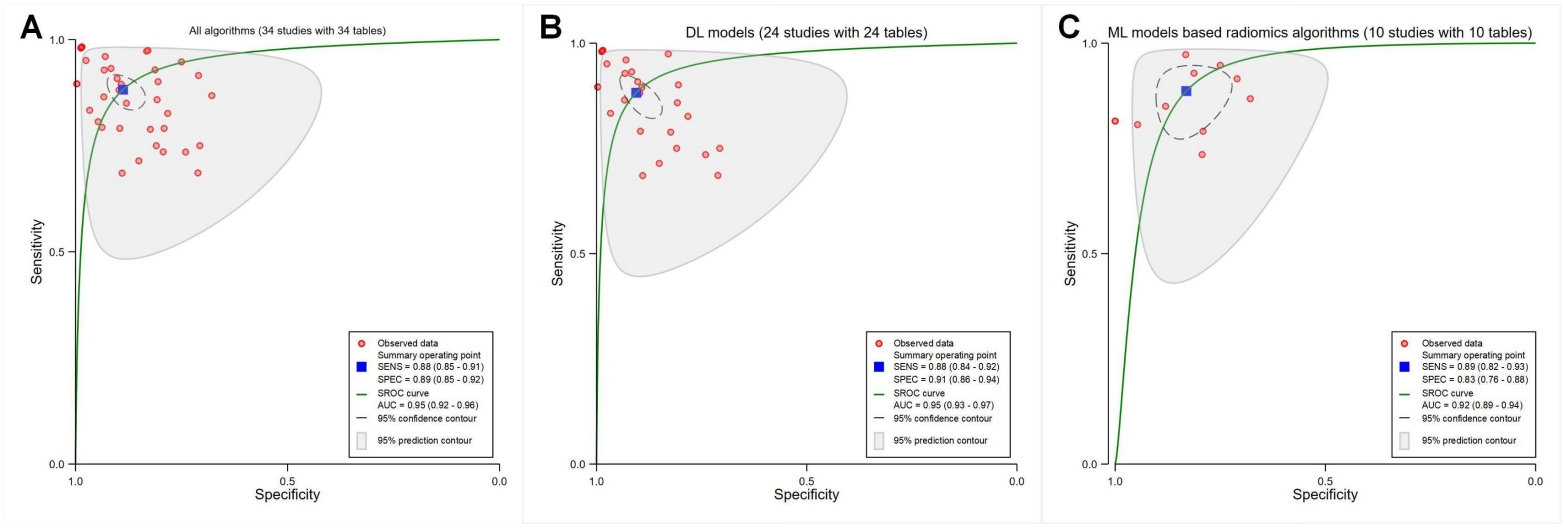
Receiver operating characteristic curves based on the overall performance of different algorithms. (A) All studies included in the meta-analysis (34 studies with 34 tables). (B) Deep learning (DL) models (24 studies with 24 tables). (C) Machine learning (ML) models based on radiomics algorithms (10 studies with 10 tables). AUC: area under the curve; SENS: sensitivity; SPEC: specificity; SROC: summary receiver operating characteristic.

The meta-analysis revealed pooled sensitivity, specificity, and an area under the SROC curve (SROC AUC) of 0.88 (95% CI 0.85‐0.91; *I*^2^=93.58%; *P*<.001; in Multimedia Appendix 2 [36-69]), 0.89 (95% CI 0.85‐0.92; *I*^2^=91.38%; *P*<.001; in Multimedia Appendix 2 [36-69]), and 0.95 (95% CI 0.92‐0.96) for all 34 studies (Figure 2A); 0.88 (95% CI 0.84‐0.92; *I*^2^=93.70%; *P*<.001; Multimedia Appendix 3 [36-69]), 0.91 (95% CI 0.86‐0.94; *I*^2^=95.55%; *P*<.001; Multimedia Appendix 3 [36-69]), and 0.95 (95% CI 0.93‐0.97) for all DL models (Figure 2B); 0.89 (95% CI 0.82‐0.93; *I*^2^=90.20%; *P*<.001; Multimedia Appendix 3 [36-69]), 0.83 (95% CI 0.76‐0.88; *I*^2^=78.92%; *P*<.001; Multimedia Appendix 3 [36-69]), and 0.92 (95% CI 0.89‐0.94) for all ML models based on radiomics algorithms (Figure 2C), respectively. Notably, some studies used multiple diagnostic models; however, the diagnostic accuracy of certain models was not thoroughly assessed.

#### Subgroup Analysis

##### Medical Imaging Modalities

**Figure 3.**
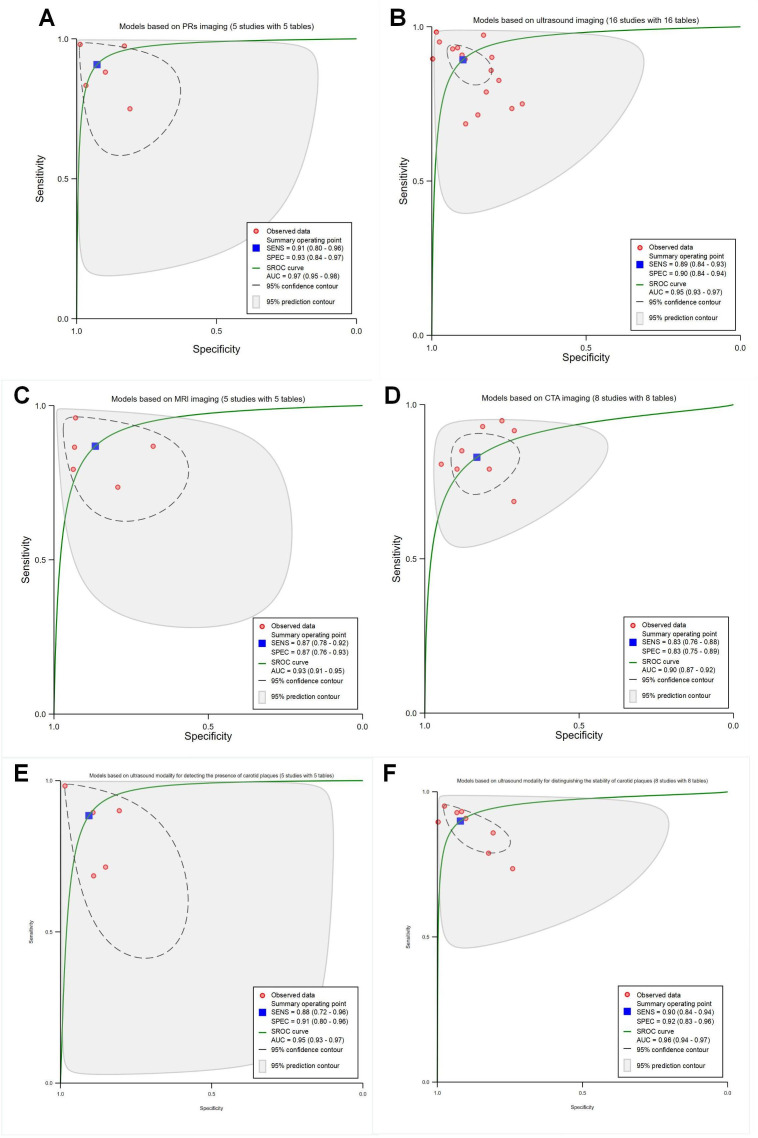
Receiver operating characteristic curves for different medical imaging modalities. (A) Periapical radiographs (PRs) imaging models (5 studies with 5 tables). (B) Ultrasound imaging models (16 studies with 22 tables). (C) Magnetic resonance imaging (MRI) models (5 studies with 7 tables). (D) Computed tomography angiography (CTA) models (8 studies with 10 tables). (E) Models based on ultrasound modality for detecting the presence of carotid plaque (5 studies with 5 tables). (F) Models based on ultrasound modality for distinguishing the stability of carotid plaques (8 studies with 8 tables). AUC: area under the curve; SENS: sensitivity; SPEC: specificity; SROC: summary receiver operating characteristic.

The pooled sensitivity, specificity, and SROC AUC were 0.91 (95% CI 0.80‐0.96), 0.93 (95% CI 0.84‐0.97), and 0.97 (95% CI 0.95‐0.98) for the 5 studies using PRs (*P*<.001; with 5 contingency tables; Figure 3A); 0.89 (95% CI 0.84‐0.93), 0.90 (95% CI 0.84‐0.94), and 0.95 (95% CI 0.93‐0.97) for the 16 studies using ultrasound images (*P*<.001with 16 contingency tables; Figure 3B); 0.87 (95% CI 0.87‐0.92), 0.87 (95% CI 0.76‐0.93), and 0.93 (95% CI 0.91‐0.95) for the 5 studies using MRI images (*P*<.001; with 5 contingency tables; Figure 3C); 0.83 (95% CI 0.76‐0.88), 0.83 (95% CI 0.75‐0.89), and 0.90 (95% CI 0.87‐0.92) for the 8 studies using CTA images (*P*<.001; with 8 contingency tables; Figure 3D), respectively. In addition, we conducted subgroup analyses using the same imaging modality based on differentiation. However, only subgroups of identifying the presence and stability of plaque had sufficient data for the ultrasound modality to perform statistical analyses and obtain pooled diagnostic performance metrics (Table S5 in Multimedia Appendix 1). The pooled sensitivity, specificity, and SROC AUC were 0.88 (95% CI 0.72‐0.96), 0.91 (95% CI 0.80‐0.96), and 0.95 (95% CI 0.93‐0.97) for determining the presence of plaques (*P*<.001; with 5 contingency tables; Figure 3E), 0.90 (95% CI 0.84‐0.94), 0.92 (95% CI 0.83‐0.96), and 0.96 (95% CI 0.94‐0.97) for distinguishing the stability of plaques (*P*<.001; with 8 contingency tables; Figure 3F).

##### Use of Transfer Learning

**Figure 4.**
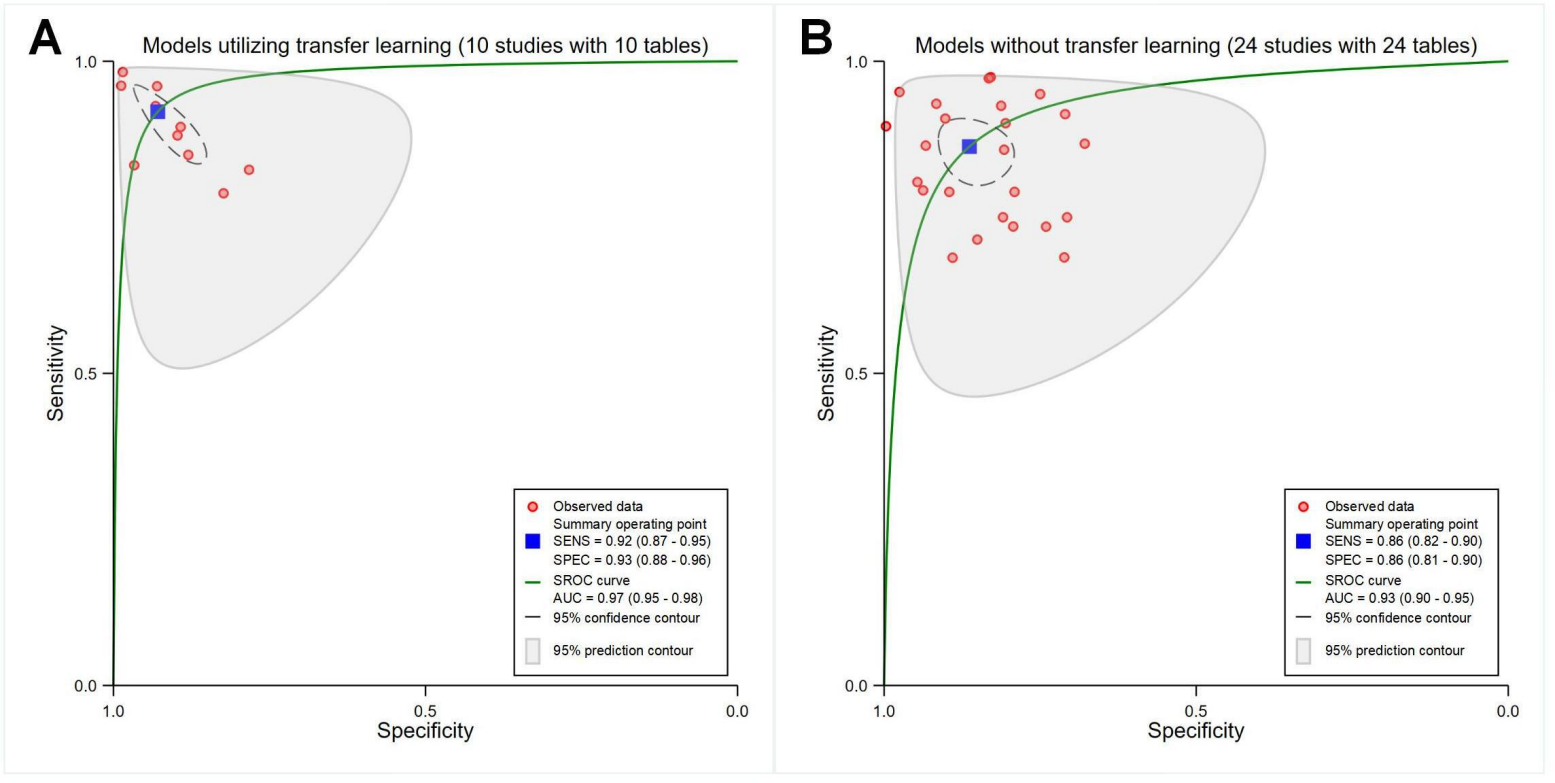
Receiver operating characteristic curves demonstrating transfer learning application. (A) Models using transfer learning (10 studies with 10 tables). (B) Models without transfer learning (24 studies with 24 tables). AUC: area under the curve; SENS: sensitivity; SPEC: specificity; SROC: summary receiver operating characteristic.

The pooled sensitivity, specificity, and SROC AUC were 0.92 (95% CI 0.87‐0.95), 0.93 (95% CI 0.88‐0.96), and 0.97 (95% CI 0.95‐0.96) for the 10 studies using transfer learning (*P*<.001; with 10 contingency tables; Figure 4A) and 0.86 (95% CI 0.82‐0.90), 0.86 (95% CI 0.81‐0.90), and 0.93 (95% CI 0.90‐0.95) for the 24 studies without transfer learning (*P*<.001; with 24 contingency tables; Figure 4B), respectively.

##### Carotid Plaque Type

**Figure 5.**
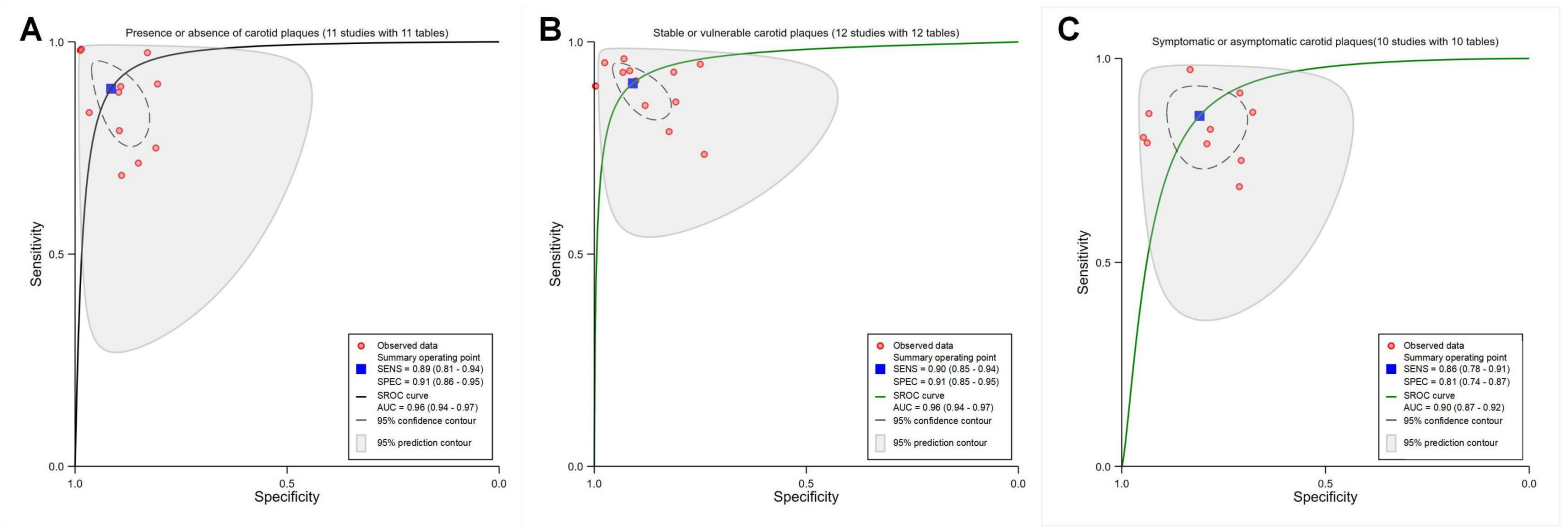
Receiver operating characteristic curves for different carotid plaque types. (A) Presence versus absence of carotid plaques (11 studies with 11 tables). (B) Stable versus vulnerable carotid plaques (12 studies with 12 tables). (C) Symptomatic versus asymptomatic carotid plaques (10 studies with 10 tables). AUC: area under the curve; SENS: sensitivity; SPEC: specificity; SROC: summary receiver operating characteristic.

The pooled sensitivity, specificity, and AUC were 0.89 (95% CI 0.81‐0.94), 0.91 (95% CI 0.86‐0.95), and 0.96 (95% CI 0.94‐0.97) for the 11 studies identifying the presence or absence of carotid plaques (*P*<.001; with 11 contingency tables; Figure 5A); 0.90 (95% CI 0.85‐0.94), 0.91 (95% CI 0.85‐0.95), and 0.96 (95% CI 0.94‐0.97) for the 12 studies identifying stable or vulnerable carotid plaques (*P*<.001; with 12 contingency tables), respectively (Figure 5B); and 0.86 (95% CI 0.78‐0.91), 0.81 (95% CI 0.74‐0.87), and 0.90 (95% CI 0.87‐0.92) for the 10 studies identifying symptomatic or asymptomatic plaques (*P*<.001; with 10 contingency tables; Figure 5C), respectively.

##### Pure Artificial Intelligence Models Versus Models Constructed by Combining Clinical Features

**Figure 6.**
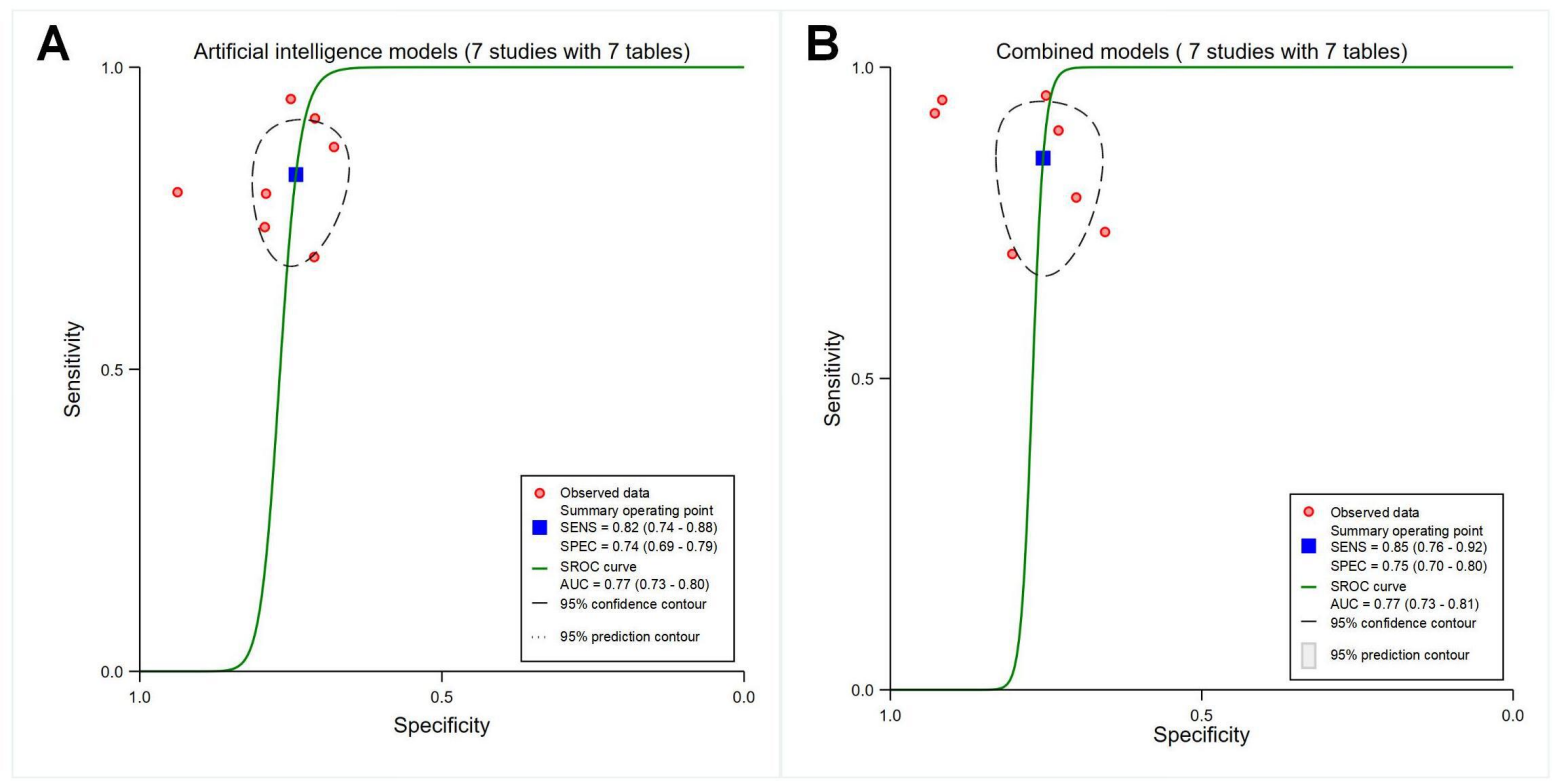
Receiver operating characteristic curves showing the diagnostic performance of pure artificial intelligence models or models constructed by combining clinical features. (A) Artificial intelligence models (7 studies with 7 tables). (B) Combined models (7 studies with 7 tables). AUC: area under the curve; SENS: sensitivity; SPEC: specificity; SROC: summary receiver operating characteristic.

The pooled sensitivity, specificity, and SROC AUC were 0.82 (95% CI 0.74‐0.88), 0.74 (95% CI 0.69‐0.79), and 0.77 (95% CI 0.73‐0.80) for the 7 studies involving pure artificial intelligence models meeting the inclusion criteria (*P*<.001; with 7 contingency tables; Figure 6A) and 0.85 (95% CI 0.76‐0.92), 0.75 (95% CI 0.70‐0.80), and 0.77 (95% CI 0.73‐0.81) for models constructed by combining clinical features (*P*<.001; with 7 contingency tables; Figure 6B), respectively.

##### Different Sets of Datasets

**Figure 7.**
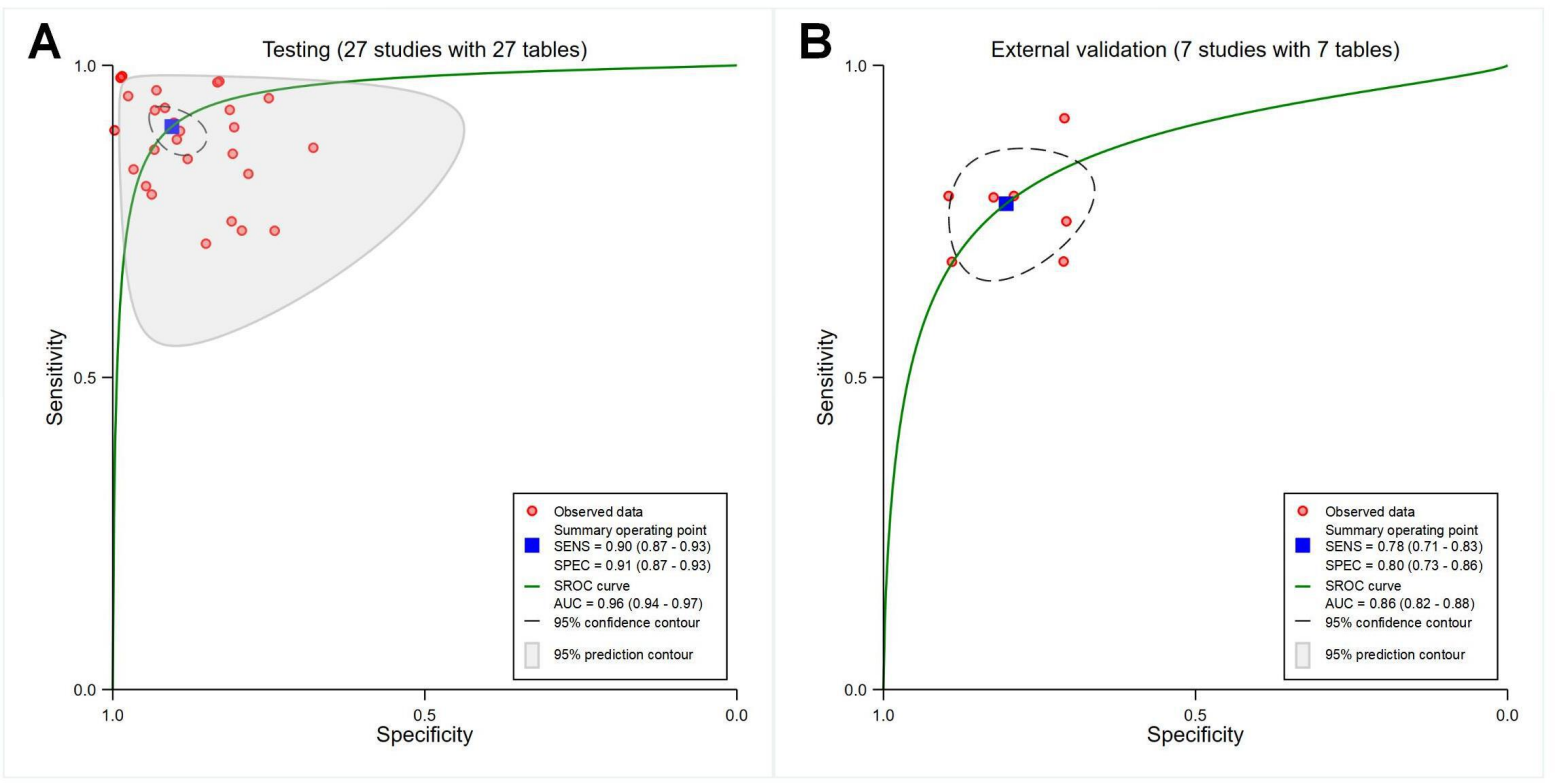
Receiver operating characteristic curves showing different sets of datasets. (A) Testing (27 studies with 27 tables). (B) External validation (7 studies with 7 tables). AUC: area under the curve; SENS: sensitivity; SPEC: specificity; SROC: summary receiver operating characteristic.

The pooled sensitivity, specificity, and AUC were 0.90 (95% CI 0.87‐0.93), 0.91 (95% CI 0.87‐0.93), and 0.96 (95% CI 0.94‐0.97) for testing sets (*P*<.001; with 27 contingency tables; Figure 7A); 0.78 (95% CI 0.71‐0.83), 0.80 (95% CI 0.73‐0.86), and 0.86 (95% CI 0.82‐0.88) for external validation sets (*P*<.001; with 7 contingency tables; Figure 7B), respectively.

##### Low and High or Unclear Risk of Bias Studies

**Figure 8.**
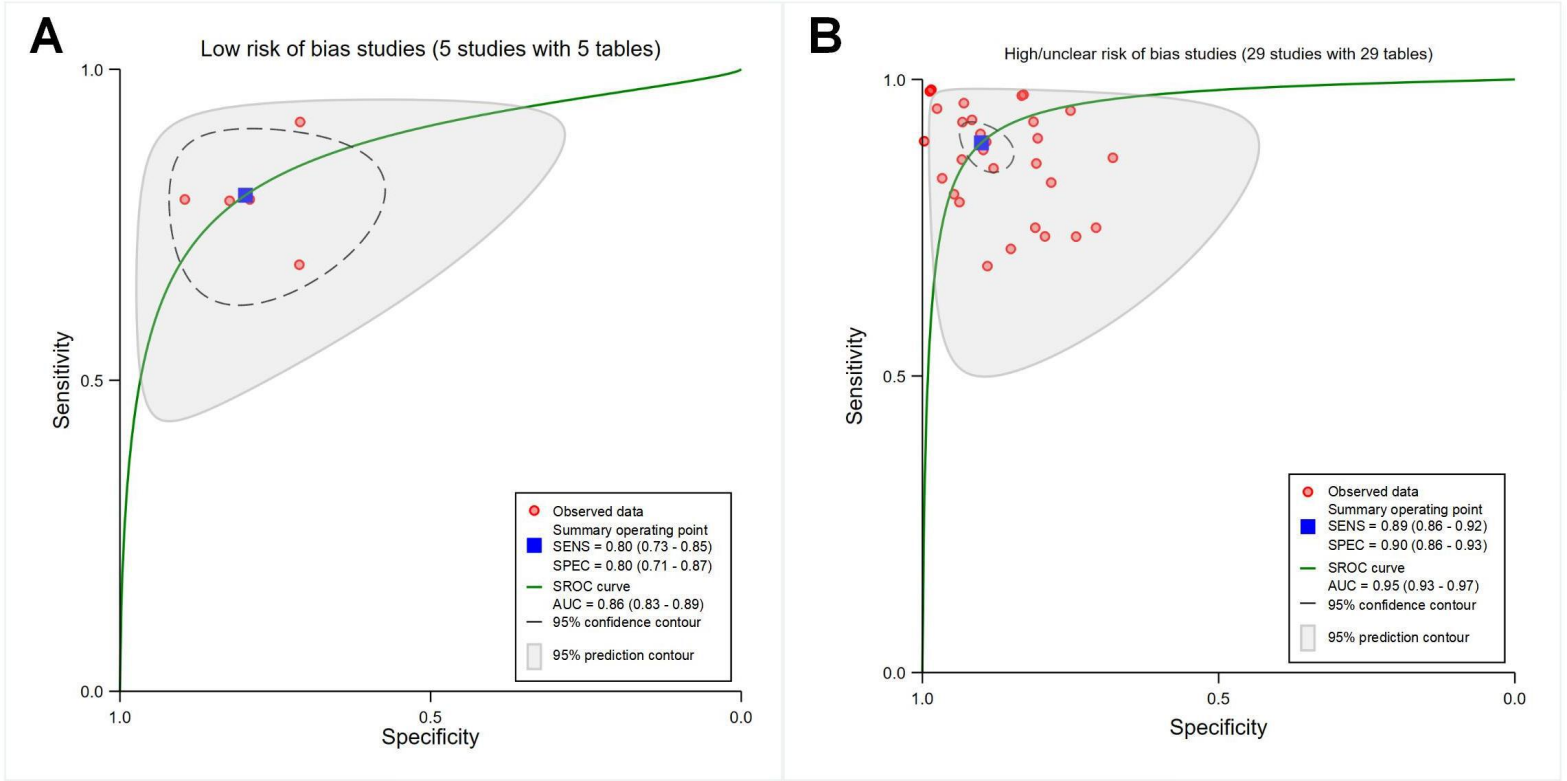
Receiver operating characteristic curves showing studies with different risk of bias. (A) Studies with a low risk of bias (5 studies with 5 tables). (B) Studies with a high/unclear risk of bias (29 studies with 29 tables). AUC: area under the curve; SENS: sensitivity; SPEC: specificity; SROC: summary receiver operating characteristic.

The pooled sensitivity, specificity, and AUC were 0.80 (95% CI 0.73‐0.85), 0.80 (95% CI 0.71‐0.87), and 0.86 (95% CI 0.83‐0.89) for studies with a low risk of bias (*P*<.001; with 5 contingency tables; Figure 8A), and 0.89 (95% CI 0.86‐0.92), 0.90 (95% CI 0.86‐0.93), and 0.95 (95% CI 0.93‐0.97) for studies with a high or unclear risk of bias (*P*<.001; with 29 contingency tables; Figure 8B), respectively.

##### Different Sample Sizes of Model

**Figure 9.**
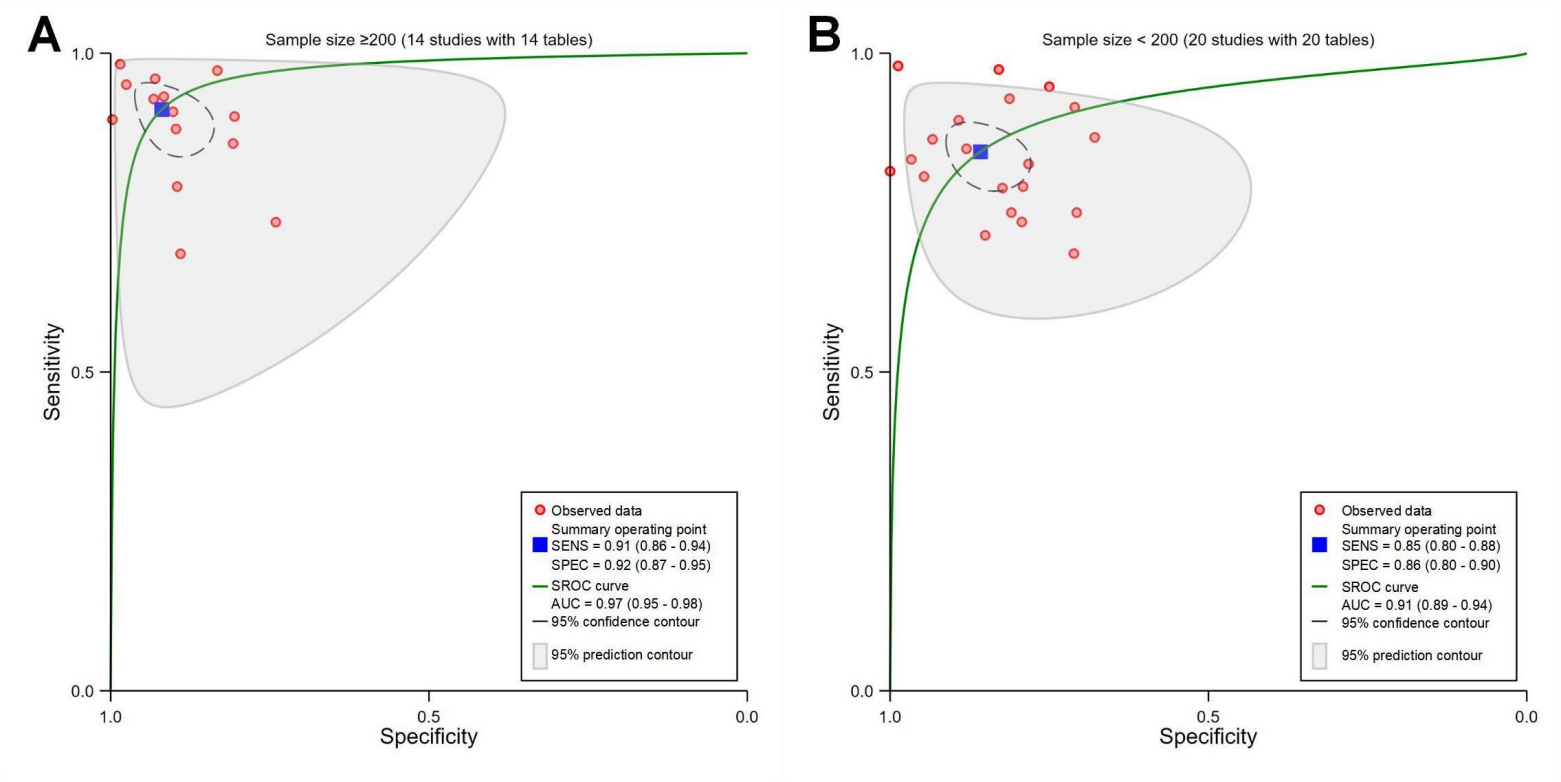
Receiver operating characteristic curves showing different sample sizes of model. (A) Sample size ≥200 (14 studies with 14 tables). (B) Sample size <200 (20 studies with 20 tables). AUC: area under the curve; SENS: sensitivity; SPEC: specificity; SROC: summary receiver operating characteristic.

The pooled sensitivity, specificity, and AUC were 0.91 (95% CI 0.86‐0.94), 0.92 (95% CI 0.87‐0.95), and 0.97 (95% CI 0.95‐0.98) for sample size≥200 (*P*<.001; with 14 contingency tables) (Figure 9A), and 0.85 (95% CI 0.80‐0.88), 0.86 (95% CI 0.80‐0.90), and 0.91 (95% CI 0.89‐0.94) for sample size<200 (*P*<.001; with 20 contingency tables; Figure 9B), respectively.

##### Models With Different Research Designs (Multicenter Studies and Single-Center Studies)

**Figure 10.**
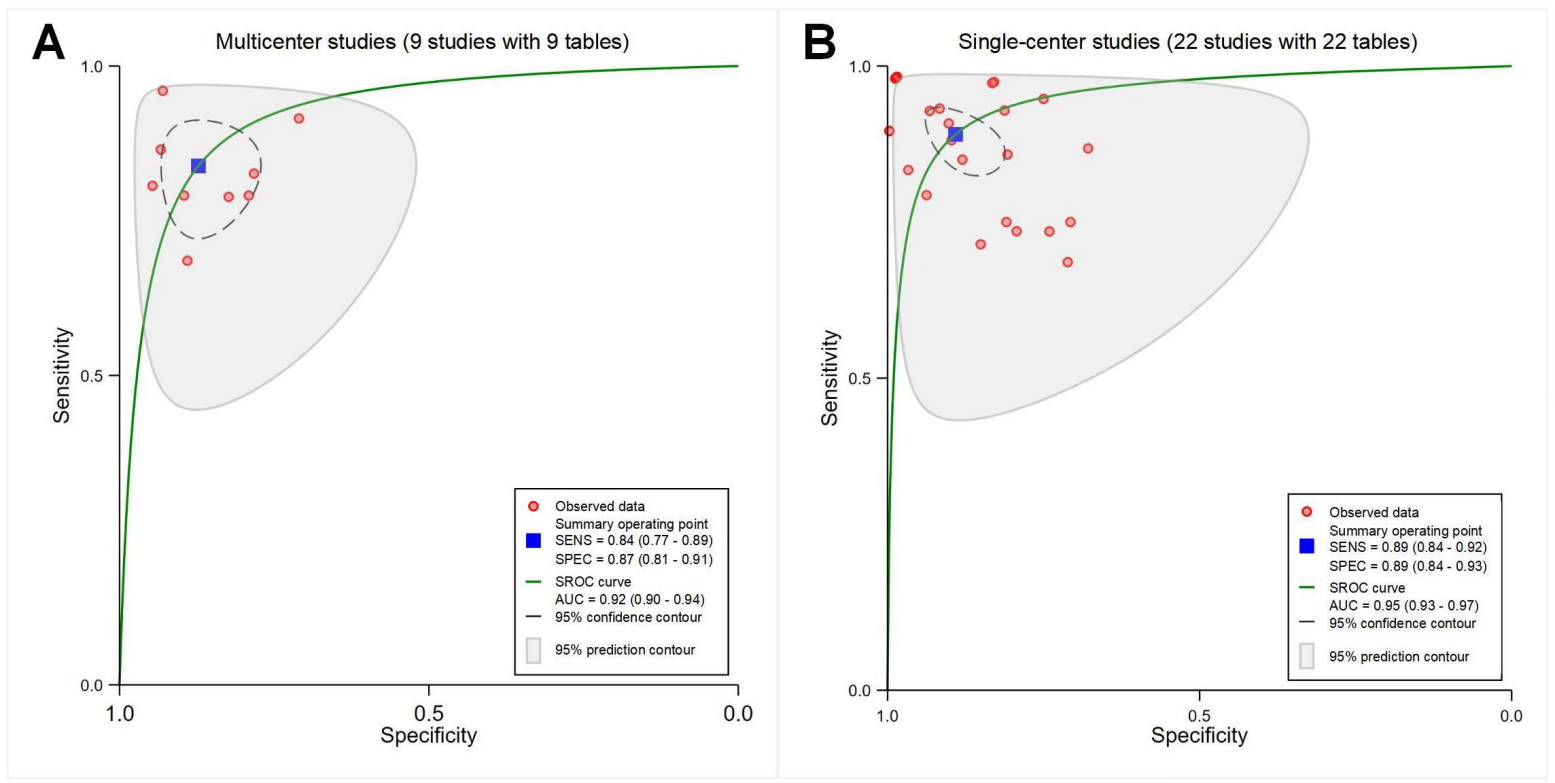
Receiver operating characteristic curves showing models with different research designs. (A) Multicenter studies (9 studies with 9 tables). (B) Single-center studies (22 studies with 22 tables). AUC: area under the curve; SENS: sensitivity; SPEC: specificity; SROC: summary receiver operating characteristic.

The pooled sensitivity, specificity, and AUC were 0.84 (95% CI 0.77‐0.89), 0.87 (95% CI 0.81‐0.91), and 0.92 (95% CI 0.90‐0.94) for multicenter studies (*P*<.001; with 9 contingency tables; Figure 10A), and 0.89 (95% CI 0.84‐0.92), 0.89 (95% CI 0.84‐0.93), and 0.95 (95% CI 0.93‐0.97) for single-center studies (*P*<.001; with 22 contingency tables; Figure 10B), respectively.

### Heterogeneity Analysis and Meta-Regression Analysis

The Cochran Q test was used to indicate the presence of heterogeneity among subgroups (significance level *P*≤.05) [15]. The *I*² index was used to assess the extent of heterogeneity among studies [15], revealing high sensitivity (*I*²=93.58%) and specificity (*I*²=91.38%; Multimedia Appendix 2). The Deek funnel plot asymmetry test, with *P*=.21, indicated no apparent publication bias (Multimedia Appendix 4). Subgroup analyses were performed using the random-effects models to identify the potential sources of heterogeneity, particularly when *I*² exceeded 50% [16]. Results were as follows:

1. AI model for carotid plaques: Both ML models based on radiomics algorithms and DL models exhibited high sensitivity, with an *I*^2^ of 90.20% and 93.70%, and high specificity, with an *I*^2^ of 78.92% and 95.55%, suggesting high performance and significant heterogeneity (Multimedia Appendix 3 [36-69]).
2. Medical imaging modalities: the sensitivity and specificity for PRs (sensitivity *I*^2^=82.28%; specificity *I*^2^=79.16%; Multimedia Appendix 5 [36-69]) and ultrasound (sensitivity *I*^2^=96.92%; specificity *I*^2^=94.98%; Multimedia Appendix 5 [36-69]). The sensitivity and specificity for MRI (sensitivity *I*^2^=71.57%; specificity *I*^2^=73.21%; Multimedia Appendix 5 [36-69]) and the sensitivity for CTA (*I*^2^=56.80%) displayed moderate heterogeneity (Multimedia Appendix 5 [36-69]). The specificity of CTA (*I*^2^=83.79%) was high (Multimedia Appendix 5 [36-69]). In the ultrasound modality, the sensitivity and specificity for determining the presence of plaques (sensitivity *I*^2^=96.78%; specificity *I*^2^=97.97%; Multimedia Appendix 5 [36-69]) and distinguishing the stability of plaques (sensitivity *I*^2^=97.01%; sensitivity *I*^2^=94.43%; Multimedia Appendix 5 [36-69]) were high.
3. Use of transfer learning: the specificity for models using transfer learning (specificity *I*^2^=74.85%; Multimedia Appendix 6 [36-69]) displayed moderate heterogeneity. The sensitivity for models using transfer learning (sensitivity *I*^2^=79.84%; Multimedia Appendix 6 [36-69]) and the sensitivity and specificity for the models without transfer learning (sensitivity *I*^2^=94.12%; specificity *I*^2^=87.35%; Multimedia Appendix 6 [36-69]) were high.
4. Carotid plaque type: all plaque types showed higher sensitivity and specificity; presence or absence of plaques (sensitivity *I*^2^=94.08%; specificity *I*^2^=97.60%; part A in Multimedia Appendix 7 [36-69]), stable or vulnerable plaques with (sensitivity *I*^2^=95.19%; specificity *I*^2^=91.29%; part B in Multimedia Appendix 7 [36-69]), and symptomatic or asymptomatic plaques (sensitivity *I*^2^=93.28%; specificity *I*^2^=84.67%; part C in Multimedia Appendix 7 [36-69]).
5. Both pure AI models and combined clinical features models did not exhibit high heterogeneity for AI models (sensitivity *I*^2^=62.97%; specificity *I*^2^=2.41%; part B in Multimedia Appendix 8 [ 50,52,58,63,64,66,68]) and combined models (sensitivity *I*^2^=69.77%; specificity *I*^2^=40.08%) for combined models (part A in Multimedia Appendix 8 [ 50,52,58,63,64,66,68]).
6. Different sets of datasets: both testing (sensitivity *I*^2^=94.23%; specificity *I*^2^=93.45%; part A in Multimedia Appendix 9 [36-69]) and external validation (specificity *I*^2^=84.42%; part B in Multimedia Appendix 9 [36-69]) were high heterogeneity, except the sensitivity for external validation (*I*^2^=66.67%; part B in Multimedia Appendix 9 [36-69]).
7. Different risk of bias studies: the sensitivity and specificity for high or unclear risk of bias studies (sensitivity *I*^2^=94.61%; specificity *I*^2^=92.59%; part B in Multimedia Appendix 10 [36-69]) and the specificity for low risk of bias studies (*I*^2^=87.10%) were high (part A in Multimedia Appendix 10 [36-69]). The sensitivity for low risk of bias studies (*I*^2^=62.20%) was moderate (part A in Multimedia Appendix 10 [36-69]).
8. Different sample sizes of model: The sensitivity and specificity for sample size ≥200 (sensitivity *I*^2^=97.91%; specificity *I*^2^=97.40%; part A in Multimedia Appendix 11 [36-69]) and the specificity for sample size <200 (*I*^2^=78.02%; part B in Multimedia Appendix 11 [36-69]) were high. The sensitivity for sample size <200 (*I*^2^=60.64%) was moderate (part B in Multimedia Appendix 11 [36-69]).
9. Models with different research designs: The sensitivity and specificity for multicenter studies (sensitivity *I*^2^=81.36%; specificity *I*^2^=80.24%; part A in Multimedia Appendix 12 [36,38,39,41-52,54-69]) and single-center studies (sensitivity *I*^2^=95.07 %; specificity *I*^2^=90.63%) were high (part B in Multimedia Appendix 12 [36,38,39,41-52,54-69]).

The meta-regression did not explore the factors contributing to heterogeneity (parts A-I in Multimedia Appendix 13 [36-69]). The results of all subgroups are depicted in Table S4 in Multimedia Appendix 1. The Fagan nomogram was used to evaluate the diagnostic performance of ML models based on radiomics algorithms and DL models for carotid plaques. The results showed a P-post of 89% and 12% for the positive and negative tests, respectively (Multimedia Appendix 14).

### Sensitivity Analysis

Excluding the specific studies did not significantly change our research results (Table S7-S8 in Multimedia Appendix 1).

### Quality Assessment

The quality of the 34 studies was evaluated using the QUADAS-AI tool (Multimedia Appendix 15). The QUADAS-AI specifically evaluates bias risk and applicability concerns in AI studies. Here, we observed that most studies had significant bias or applicability concerns, particularly regarding the selection of patients and index test. In the “patient selection” domain, 20 studies were classified as either high-risk or indeterminate due to reliance on closed-access data or failure to present the rationale and breakdown of its training, validation, and test sets. Only 7 externally validated studies were classified as low-risk in the “index test” category, while others showed elevated risks due to a lack of validation. In the “reference standard” assessment, the reference standard of all studies could be used to classify the target condition correctly. For the “flow and timing” assessment, 10 studies showed indeterminate risks due to insufficient justification for the timing between index and reference tests. Additionally, 20 studies presented significant concerns regarding applicability in the “patient selection” domain, receiving unclear ratings. In the “index test” domain, 7 studies were rated as having low applicability, while all studies received low applicability ratings in the “Reference Standard” domain.

## Discussion

### Principal Findings

This study represents the first systematic evaluation of ML models based on radiomics and DL models for the characterization of extracranial carotid plaques. Both approaches demonstrated robust diagnostic performance, with high SROC values of 0.95 and 0.92, respectively, highlighting their promising potential for clinical application in plaque detection and risk stratification.

Initially, the SP and SROC AUC of DL models were improved compared to ML models based on radiomics (0.91 vs 0.83; 0.95 vs 0.92), while their sensitivity was similar to that of ML (0.88). Moreover, we observed that radiomics and DL models used to identify the presence of plaques and stable plaques had similar diagnostic capabilities (SROC 0.96, 95% CI 0.94‐0.97), and both were effective in identifying symptomatic plaques (SROC 0.90, 95% CI 0.87‐0.92). Notably, these differences may not be simply due to model performance, but could result from a combination of different clinical objectives (simple exclusion diagnosis or differentiation of specific cases), imaging variations, and model techniques. By using knowledge gained from previous tasks, transfer learning enhances model performance on new datasets and minimizes data requirements. It has been successfully applied in various areas of cardiovascular disease to boost the performance of models [2,76,77]. In subgroup analyses, transfer learning significantly enhances model performance in data-limited scenarios and prevents overfitting. Large sample sizes can minimize sampling bias, decrease overfitting, and enhance the stability and reproducibility of the models. Moreover, we performed more detailed subgroup analyses based on the same imaging modality. Only the type of plaques in the ultrasound modality had sufficient data to perform statistical analysis and obtain summary diagnostic efficacy indicators. Results showed that ultrasound-based models have demonstrated excellent and similar performance in detecting the presence of plaques and assessing their stability. Considering the differences in equipment characteristics, patient demographics, and study design, these findings should be interpreted with caution. Nevertheless, these results provide valuable insights into the efficacy of radiomics algorithms and DL models in the diagnosis of carotid plaque.

### Analysis of the Main Aspects

This meta-analysis demonstrates that radiomics-based models and DL models can diagnose extracranial carotid plaque, but the advantages of DL models in specificity and SROC should be interpreted with caution. A review of the included studies revealed that, among the 24 investigations using DL models, 20 primarily focused on plaque characterization (11 on the detection of plaques and 9 on plaque stability). Of these, 13 studies used ultrasound imaging to identify plaque-specific features such as echogenicity, morphology, and composition. In contrast, among the 10 studies using radiomics-based ML models, 6 were dedicated to identifying symptomatic plaques, predominantly using MRI (n=2) and CTA (n=3). The accuracy of symptomatic plaque identification was influenced not only by intrinsic imaging characteristics but also by clinical indicators, including plaque rupture, thrombus formation, and the occurrence of cerebral hypoperfusion. The tasks were more complex, and model training seemed to focus on reducing false negatives to lower the risk of adverse outcomes such as stroke. In addition, traditional ML algorithms may rely on manual preprocessing and struggle to capture other subtle differences (such as the presence of tiny thrombi or fibrous cap thickness), which may introduce variability and additional costs. In contrast, the DL models (particularly convolutional neural networks) do not rely on artificially designed features; instead, they can directly process raw medical images, automatically filter noise, and automatically extract more meaningful image features (eg, slight echo attenuation behind plaques, differences in vascular wall elasticity, etc) [78]. It can also analyze the preset artificial extraction features, conduct independent learning, and uncover potential rules, thereby addressing the aforementioned challenges [23,79]. It is worth noting that a mismatch in the number of studies may also affect the interpretation of the results. Therefore, these differences may not be simply due to model performance, but could also be caused by multiple factors, which need to be further investigated.

Besides, the “black box” nature of AI algorithms, particularly DL models, raises concerns about the transparency and reliability of decision-making. Of the 34 studies reviewed, only 2 used explainable DL models, achieving an accuracy of 98.2% [37,65]. The explainable AI (XAI) approach leverages visualization techniques, feature attribution analysis, and both global and local explanations to clarify how models derive predictions from input data. By enhancing transparency, XAI fosters greater trust among medical professionals, strengthens model reliability and accountability, and helps mitigate concerns related to opaque decision-making [80]. The integration of XAI in medicine not only represents a technological advancement but also ensures safe, efficient, and robust medical decision-making, which needs to be further investigated. To realize this potential, a clinically oriented XAI implementation framework needs to be developed. First, the reporting criteria for interpretable techniques (including clinical applicability evaluation and operational guidelines) should be standardized to lower the threshold for physician use. Second, the design of algorithms should be optimized through collaborative efforts of medical professionals and engineers to improve the specificity of feature attribution methods based on real clinical needs. Further clinical validation studies are needed to evaluate the practical utility of XAI across diverse diagnostic settings—such as varying regions, hospital levels, and clinician experience—and to determine its true value in supporting clinical decision-making beyond algorithmic performance [28]. Furthermore, incomplete disclosure of model development processes in reports, selective presentation of results by investigators, and heterogeneity in diagnostic standard implementation across practitioners with different levels of experience may decrease the reliability and generalizability of findings. Therefore, we recommend the formulation of standardized imaging protocols, reporting procedures, and quality control measures for carotid plaque assessment and advocate for the establishment of specialized AI reporting guidelines for cardiovascular diseases.

Advances in imaging technology have now largely met the diagnostic requirements of current clinical practice, and current guidelines place heavy reliance on imaging tests for carotid plaque assessment. Among the 34 included studies, 27 constructed diagnostic models based only on imaging data. However, this should not be interpreted as rendering other clinical parameters irrelevant. Multidimensional diagnostic models combined with clinical features have been shown to achieve good diagnostic performance in identifying various diseases, such as pancreatic ductal adenocarcinoma [81], HCC recurrence after liver transplantation [82], hemorrhagic brain metastases [83], malignant BI-RADS 4 breast masses [84], and others. In our study, the diagnostic performance of combined models did not slightly improve, which may be due to the small sample size or some features could not provide more diagnostic information (for example, Hu et al [2] constructed a model relying only on indirect perivascular adipose tissue radiomic features and clinical features to identify symptomatic plaques, lacking direct imaging features). Considering this evidence, we strongly recommend that future research should aim to not only systematically incorporate laboratory tests, medical history, and other clinical parameters to develop multidimensional diagnostic models, but also to summarize the most meaningful features for specific types of plaques. This could address the limitations in current studies regarding single imaging modalities. This will also improve the precise classification of carotid plaques and personalized risk assessment.

This meta-analysis identified significant heterogeneity, while meta-regression and subgroup regression analysis did not identify the source, primarily attributable to the intrinsic challenges in regulating all potential confounding factors. Different imaging techniques can affect model performance based on the type of images used (static images vs dynamic videos), the equipment, and the operators. Guang et al [57] used a contrast-enhanced ultrasound video-based DL model to evaluate the diagnostic efficacy of a new carotid network structure for assessing carotid plaques, whereas other ultrasound studies consistently used static images. The sequence of MRI scans also influences diagnostic outcomes. Zhang et al [58] reported that a model incorporating a combination of T1-weighted, T2-weighted, dynamic contrast-enhanced, and postcontrast (POST) MRI sequences achieved a higher AUC for identifying high-risk carotid plaques compared to models using individual sequences or partial combinations. This enhanced performance is attributed to the complementary nature of these imaging sequences, each capturing distinct pathophysiological characteristics of the plaque, thereby improving diagnostic accuracy when used in combination. PRs have limited resolution, only detecting calcified components of carotid plaques and missing features such as lipid-rich necrotic cores or thin or ruptured fibrous caps. There are also notable differences in model architecture. Yoo et al [39] found performance variations among different convolutional neural network architectures within the CACSNet framework on the same dataset. Gui et al [49] compared multiple DL models (eg, 3D-DenseNet, 3D-SE-DenseNet) with 9 ML algorithms (including Decision Tree, Random Forest, SVM, etc) using identical datasets. They found that DL models generally performed better across key metrics like AUC and accuracy, with significant performance differences between and within the two model types. These suggest that scanning parameters, model architectures, image segmentation, and algorithms may explain the heterogeneity in the research results. However, the small number of studies limits our ability to perform comprehensive subgroup analyses, which need to be further investigated.

The use of AI has significantly promoted the diagnosis of carotid plaque; however, its application requires cautious evaluation. Only 9 studies were multicenter (most used external validation), with diagnostic performance lower than single-center studies. Most studies (n=29) had a high risk of bias due to a lack of open-source data and external validation and failure to present the rationale and breakdown of its sets, which led to overestimation of the research results and affected the reproducibility and generalizability of the findings. Similar issues have been noted in previous reports, highlighting a broader deficiency in rigorous research standards within the field [85-87]. Furthermore, the contingency tables mostly come from the testing sets. Although the testing set achieved the best diagnostic performance, it had higher data quality or similar data distribution to the training, or overfitting noise, resulting in inaccurate performance estimation, and strong regularization may also decrease its performance, ultimately undermining clinical confidence in these models.

This study has certain clinical significance. We conducted an in-depth literature review and methodological quality evaluation, presenting the most current and comprehensive systematic review of AI-based diagnostic approaches for assessing carotid plaque. The findings reveal that AI technology shows considerable potential for diagnosing carotid plaque, but the findings need to be further validated by conducting more rigorous external validation using large-scale, high-quality independent datasets.

### Limitations

This study has several limitations. First, the heterogeneity in model architectures and validation methods across studies prevents definitive conclusions regarding the most effective AI approaches. Second, many studies lack multicenter external validation, leading to a high risk of bias. The model overfitting and clinical applicability need to be carefully evaluated. Third, meta-regression and subgroup analysis did not identify the sources of high heterogeneity that existed in most of the included studies. We hypothesize that this heterogeneity may be caused by scanning parameters, model architectures, image segmentation, and algorithms. However, the overly scattered distribution of subgroups due to the limited number of studies restricts more in-depth subgroup analyses. Finally, although the Deeks test did not show significant publication bias, the included studies may have intentionally unreported negative results and omitted potentially relevant non-English literature.

Future studies should use a more comprehensive analytical methodology based on the current model. Researchers should strictly follow regulatory norms and standardized operating procedures. Prospective and multicenter studies and additional external validation are warranted to enhance the robustness and generalizability of the existing models. In the future, researchers should perform independent systematic reviews on specific subtopics—such as imaging modalities, lesion types, or model architectures—to facilitate targeted evaluations of AI performance across distinct clinical scenarios. In addition, studies on imaging modalities such as CT and MRI are advocated to generate more data, conduct subgroup analyses, and clarify the optimal matching of modality, plaque type, and algorithm. Future efforts should focus on identifying more meaningful features and building and evaluating the diagnostic performance of multidimensional diagnostic models. In parallel, establishing clinically oriented, XAI frameworks will be essential for enhancing transparency.

### Conclusions

Current findings indicate that radiomics algorithms and DL models can effectively diagnose extracranial carotid plaque. However, the irregularities in research design and the lack of multicenter studies and external validation limit the robustness of the present findings. Future research should aim to reduce bias risk and enhance the generalizability and clinical orientation of the models.

## Supplementary material

10.2196/77092 Multimedia Appendix 1 Complete supplementary data tables for the systematic review and meta-analysis.

10.2196/77092 Multimedia Appendix 2 Combined diagnostic performance estimates from the included studies (34 studies with 34 tables) [36-69].

10.2196/77092 Multimedia Appendix 3 Sensitivity and specificity of deep learning (DL) versus radiomics-based machine learning (ML) models. (A) DL models (24 studies with 24 tables). (B) ML models based on radiomics algorithms (10 studies with 10 tables) [36-69].

10.2196/77092 Multimedia Appendix 4 Publication bias.

10.2196/77092 Multimedia Appendix 5 Sensitivity and specificity for different medical imaging modalities. (A) Models based on periapical radiographs (PRs) imaging (5 studies with 5 tables). (B) Models based on ultrasound imaging (16 studies with 16 tables). (C) Models based on magnetic resonance imaging (MRI) imaging (5 studies with 5 tables). (D) Models based on computed tomography angiography (CTA) imaging (8 studies with 8 tables). (E) Models based on ultrasound modality for detecting the presence of carotid plaque (5 studies with 5 tables). (F) Models based on ultrasound modality for distinguishing the stability of carotid plaques (8 studies with 8 tables) [36-69].

10.2196/77092 Multimedia Appendix 6 Sensitivity and specificity of models for using transfer learning or not. (A) Models using transfer learning (10 studies with 10 tables). (B) Models without transfer learning (24 studies with 24 tables) [36-69].

10.2196/77092 Multimedia Appendix 7 Sensitivity and specificity for different diagnostic tasks. (A) Presence or absence of carotid plaques (11 studies with 11 tables). (B) Stable or vulnerable carotid plaques (12 studies with 12 tables). (C) Symptomatic or asymptomatic carotid plaques (10 studies with 11 tables) [36-69].

10.2196/77092 Multimedia Appendix 8 Sensitivity and specificity of pure artificial intelligence models or models constructed by combining clinical features for carotid plaques. (A) Combined models (7 studies with 7 tables). (B) Artificial intelligence models (7 studies with 7 tables) [50,52,58,63,64,66,68].

10.2196/77092 Multimedia Appendix 9 Sensitivity and specificity for different dataset types. (A) Testing (27 studies with 27 tables). (B) External validation (7 studies with 7 tables) [36-69].

10.2196/77092 Multimedia Appendix 10 Sensitivity and specificity for different risk of-bias studies. (A) Low risk of bias studies (5 studies with 5 tables). (B) High/unclear risk of bias studies (29 studies with 29 tables) [36-69].

10.2196/77092 Multimedia Appendix 11 Sensitivity and specificity of study using different sample sizes. (A) Sample size ≥200 (14 studies with 14 tables). (B) Sample size <200 (20 studies with 20 tables) [36-69].

10.2196/77092 Multimedia Appendix 12 Sensitivity and specificity for different research designs. (A) Multicenter studies (9 studies with 9 tables). (B) Single-center studies (22 studies with 22 tables) [36,38,39,41-52,54-69].

10.2196/77092 Multimedia Appendix 13 Exploration of potential sources of heterogeneity across multiple variables. (A) Different algorithms (deep learning models or machine learning models based radiomics algorithms). (B) Different medical imaging modalities. (C) Different carotid plaque type. (D) Utilizing transfer learning or not. (E) Different sets of datasets. (F) Different sample size. (G) Single-center or multicenter studies. (H) Pure artificial intelligence (AI) models or models constructed by combining clinical features. (I) Different risk of bias studies [36-69].

10.2196/77092 Multimedia Appendix 14 The Fagan nomogram assesses the diagnostic ability of radiomics and deep learning to carotid plaques.

10.2196/77092 Multimedia Appendix 15 Quality Assessment of Diagnostic Accuracy Studies for Artificial Intelligence (QUADAS-AI) for the assessment of the methodological qualities of all the enrolled studies.

10.2196/77092 Checklist 1 PRISMA 2020 checklist.

## Abbreviations

AI: artificial intelligence
AUC: area under the curve
BI-RADS: Breast Imaging Reporting and Data System
CTA: computed tomography angiography
DL: deep learning
IEEE: Institute of Electrical and Electronics Engineers
LR: likelihood ratio
ML: machine learning
MRI: magnetic resonance imaging
P-post: posttest probability
PR: periapical radiograph
PRISMA: Preferred Reporting Items for Systematic Reviews and Meta-Analyses
PRISMA-DTA: Preferred Reporting Items for Systematic Reviews and Meta-Analyses of Diagnostic Test Accuracy Studies
PROSPERO: International Prospective Register of Systematic Reviews
QUADAS-2: Quality Assessment of Diagnostic Accuracy Studies 2
QUADAS-AI: Quality Assessment of Diagnostic Accuracy Studies for Artificial Intelligence
ROC: receiver operating characteristic
SROC: summary receiver operating characteristic
SROC AUC: area under the summary receiver operating characteristic curve
XAI: explainable AI
