# Supplementary material for: Diagnostic Performance of Deep Learning and Radiomics in Extracranial Carotid Plaque Detection: Systematic Review and Meta-Analysis
Source: J Med Internet Res. 2026 Jan 22;28:e77092. doi: 10.2196/77092 (PMC12826653; doi:10.2196/77092)
Supplement: Multimedia Appendix 1 [file jmir-v28-e77092-s001.doc]

Table S1. Search strategies.

| **Database** | **Search strategy** | **Results** |
| --- | --- | --- |
| Pubmed (n=1268) | #1 | "artificial intelligence"[Mesh] OR "radiomics"[Mesh] OR "deep learning"[Mesh] OR "neural networks, computer"[Mesh] OR "machine learning"[Mesh] |
|  | #2 | (artificial intelligence*[Title/Abstract]) OR (radiomics*[Title/Abstract]) OR (deep learning*[Title/Abstract]) OR (neural networks*[Title/Abstract]) OR (machine learning*[Title/Abstract]) OR (decision tree[Title/Abstract]) OR (random forest[Title/Abstract]) OR (bayesian learning[Title/Abstract]) OR (support vector machine[Title/Abstract]) OR (nearest neighbor[Title/Abstract]) |
|  | #3 | #1 OR #2 |
|  | #4 | "carotid artery diseases"[MeSH] OR "carotid stenosis"[MeSH] OR "Plaque, Atherosclerotic*"[Mesh] |
|  | #5 | (carotid artery diseases*[Title/Abstract]) OR (carotid stenosis*[Title/Abstract]) OR (plaque*[Title/Abstract]) OR (atherosclerotic*[Title/Abstract]) OR (carotid artery disorder*[Title/Abstract]) OR (carotid arterial disease*[Title/Abstract]) OR (carotid arterial disorder*[Title/Abstract]) OR (carotid atherosclerotic*[Title/Abstract]) OR (carotid artery stenosis*[Title/Abstract]) OR (carotid ulcer*[Title/Abstract]) OR (carotid artery obstruction*[Title/Abstract]) OR (carotid artery constriction*[Title/Abstract]) OR (carotid arterial obstruction*[Title/Abstract]) OR (carotid arterial constriction*[Title/Abstract]) OR (atheroma[Title/Abstract]) |
|  | #6 | #4 OR #5 |
|  | #7 | "sensitivity and specificity"[Mesh] OR "area under curve"[Mesh] OR "ROC curve"[Mesh] |
|  | #8 | (sensitivity and specificity[Title/Abstract]) OR (calibrat*[Title/Abstract]) OR (area under the curve[Title/Abstract]) OR (ROC[Title/Abstract]) OR (AUC[Title/Abstract]) OR (goodness of fit[Title/Abstract]) OR (performance[Title/Abstract]) OR (accuracy*[Title/Abstract]) |
|  | #9 | #7 OR #8 |
|  | #10 | #3 AND #6 AND #9 |
| Embase (n=2259) | #1 | 'artificial intelligence'/exp OR 'radiomics*'/exp OR 'machine learning'/exp OR 'deep learning'/exp OR 'neural networks'/exp OR 'decision tree'/exp OR 'random forest'/exp OR 'nearest neighbor'/exp OR 'support vector machine'/exp OR 'bayesian learning/exp |
|  | #2 | 'carotid artery disease'/exp OR 'carotid stenosis'/exp OR 'plaque*'/exp OR 'atherosclerotic*'/exp OR 'carotid artery disorder'/exp OR 'carotid arterial disorder'/exp OR 'carotid atherosclerotic'/exp OR 'carotid artery stenosis'/exp OR 'carotid ulcer'/exp OR 'carotid artery obstruction'/exp OR 'carotid artery constriction'/exp OR 'carotid arterial obstruction'/exp OR 'carotid arterial constriction'/exp |
|  | #3 | 'performance'/exp OR 'sensitivity'/exp OR specificity'/exp OR 'diagnostic accuracy'/exp OR 'area under the curve'/exp OR 'goodness of fit' OR 'calibrat*' OR 'AUC' OR 'ROC' |
|  | #4 | #1 AND #2 AND #3 |
| Cochrane Library (n=69) | #1 | MeSH descriptor: [carotid artery diseases] explode all trees |
|  | #2 | MeSH descriptor: [Carotid Stenosis] explode all trees |
|  | #3 | MeSH descriptor: [Plaque, Atherosclerotic] explode all trees |
|  | #4 | (carotid artery diseases):ti,ab,kw OR (carotid stenosis):ti,ab,kw OR ('plaque):ti,ab,kw OR (atherosclerotic):ti,ab,kw OR (carotid artery disorder):ti,ab,kw OR (carotid arterial disorder):ti,ab,kw OR (carotid atherosclerotic):ti,ab,kw OR (carotid artery stenosis):ti,ab,kw OR (carotid ulcer):ti,ab,kw OR (carotid ulcer):ti,ab,kw OR (carotid artery obstruction):ti,ab,kw OR (carotid artery constriction):ti,ab,kw OR (carotid arterial obstruction):ti,ab,kw OR (carotid arterial constriction):ti,ab,kw |
|  | #5 | #1 OR #2 OR #3 OR #4 |
|  | #6 | MeSH descriptor: [Deep Learning] explode all trees |
|  | #7 | MeSH descriptor: [Radiomics] explode all trees |
|  | #8 | MeSH descriptor: [Artificial Intelligence] explode all trees |
|  | #9 | MeSH descriptor: [Neural Networks, Computer] explode all trees |
|  | #10 | MeSH descriptor: [Machine Learning] explode all trees |
|  | #11 | (artificial intelligence):ti,ab,kw OR (radiomics):ti,ab,kw OR (deep learning):ti,ab,kw OR (neural network):ti,ab,kw OR (machine learning):ti,ab,kw OR (decision tree):ti,ab,kw OR (random forest):ti,ab,kw OR (bayesian learning):ti,ab,kw OR (support vector machine):ti,ab,kw OR (nearest neighbor):ti,ab,kw |
|  | #12 | #6 OR #7 OR #8 OR #9 OR #10 OR #11 |
|  | #13 | MeSH descriptor: [Sensitivity and Specificity] explode all trees |
|  | #14 | MeSH descriptor: [Area Under Curve] explode all trees |
|  | #15 | MeSH descriptor: [ROC Curve] explode all trees |
|  | #16 | (performance):ti,ab,kw OR (calibrat):ti,ab,kw OR (AUC):ti,ab,kw OR (ROC):ti,ab,kw OR (accuracy):ti,ab,kw OR (area under curve):ti,ab,kw OR (sensitivity):ti,ab,kw OR (specificity):ti,ab,kw |
|  | #17 | #13 OR #14 OR #15 OR #16 |
|  | #18 | #5 AND #11 AND #17 |
| Web of Science (n=233) | #1 | carotid artery diseases (Topic) OR carotid artery disease (Topic) OR carotid artery disorder (Topic) OR carotid arterial disease (Topic) OR carotid arterial disorder (Topic) OR carotid atherosclerotic (Topic) OR carotid disease (Topic) OR carotid stenosis (Topic) OR carotid artery narrowing (Topic) OR carotid artery stenosis (Topic) OR carotid ulcer (Topic) OR carotid artery obstruction (Topic) OR carotid artery constriction (Topic) OR plaque (Topic) OR atherosclerotic plaque (Topic) OR atheroma (Topic) |
|  | #2 | artificial intelligence (Topic) OR machine learning (Topic) OR deep learning (Topic) OR DL (Topic) OR ML (Topic) OR neural network (Topic) OR radiomics (Topic) OR decision tree (Topic) OR random forest (Topic) OR bayesian learning (Topic) OR support vector machine (Topic) OR nearest neighbor (Topic) |
|  | #3 | performance (Topic) OR calibrat (Topic) OR AUC (Topic) OR ROC (Topic) OR accuracy (Topic) OR area under curve (Topic) OR sensitivity (Topic) OR specificity (Topic) |
|  | #4 | #1 AND #2 AND #3 |
| IEEE (n=2005) | #1 | (Full Text & Metadata:artificial intelligence) OR (Full Text & Metadata:machine learning) OR (Full Text & Metadata:deep learning) OR (Full Text & Metadata: DL ) OR (Full Text & Metadata:ML) OR (Full Text & Metadata:neural network) OR (Full Text & Metadata:radiomics) OR (Full Text & All Metadata:decision tree) OR (Full Text & All Metadata:random forest) OR (Full Text & All Metadata:bayesian learning) OR (Full Text & All Metadata:nearest neighbor) OR (Full Text & All Metadata: support vector machine) |
|  | #2 | (Full Text & Metadata:carotid artery diseases) OR (Full Text & Metadata:carotid artery disease) OR (Full Text & Metadata:carotid artery disorder) OR (Full Text & Metadata: carotid arterial disease) OR (Full Text & Metadata:carotid arterial disorder) OR (Full Text & Metadata:carotid atherosclerotic) OR (Full Text & Metadata:carotid disease) OR (Full Text & Metadata:carotid stenosis) OR (Full Text & Metadata:carotid artery narrowing OR (Full Text & Metadata:carotid artery stenosis) OR (Full Text & Metadata:carotid ulcer) OR (Full Text & Metadata:carotid artery obstruction) OR (Full Text & Metadata:carotid arterial constriction) OR (Full Text & Metadata:plaque) OR (Full Text & Metadata:atherosclerotic plaque) OR (Full Text & Metadata: atheroma Topic) |
|  | #3 | (Full Text & Metadata:performance) OR (Full Text & Metadata:sensitivity) OR (Full Text & Metadata:specificity) OR (Full Text & Metadata:accuracy) OR (Full Text & Metadata:area under the curve) OR (Full Text & Metadata:AUC) OR (Full Text & Metadata:ROC) OR (Full Text & Metadata: area under curve) OR (Full Text & Metadata:calibrat) |
|  | #4 | #1 AND #2 AND #3 |

Table S2. Selection criteria of diagnostic modelling studies in PICOTS format

|  | **Participants (P)** | **Intervention (I)** | **Control (C)** | **Outcomes (O)** | **Timeframe (T)** | **Setting (S)** | **Other Criteria** |
| --- | --- | --- | --- | --- | --- | --- | --- |
| **Inclusion criteria** | Adults (≥18 years) subjects with Adults subject with suspected or confirmed extracranial carotid plaques, including studies focused on: plaque detection, differentiation of unstable or symptomatic plaques.  . | AI-based diagnostic models using:  Deep learning (CNN, Transformer, GAN, etc.);  Radiomics algorithms(handcrafted or deep feature extraction);  Hybrid approaches  Applied to medical imaging. | Reference standard: Ultrasound/CTA/MRA/DSA/histopathology; Alternative diagnostic methods (e.g., human expert assessment). | Diagnostic performance metrics sufficient for meta-analysis: Reported or derivable quantitative measures including true positives (TP), false positives (FP), true negatives (TN), false negatives (FN), or directly calculable indices (sensitivity, specificity, AUC, etc.).  Model robustness: Calibration curves  Technical metrics: ROC curves | before December, 2024 | Clinical diagnostic studies:  Prospective/retrospective cohorts；Diagnostic accuracy trials；Model validation studies； Model development/validation studies；Comparative studies | Full-text publications in English |
| **Exclusion criteria** | Animal studies;  Intracranial/coronary plaques;  Pediatric populations (<18 years); | Diagnostic modelling without an explicit DL or radiomics algorithms;  Only focus on research on image segmentation or image feature extraction methods.  Predictive models. | Studies without reference standard |  |  | Informal publication types (e.g., reviews, letters to the editor, editorials, conference abstracts).  . | Other language  Studies that did not report validation/test sets. |

**Table S3. Baseline characteristics of included 40 studies for the meta-analysis**

| **First author, year** | **Sex composition** | **Source of data** | **Reference standard** | **Type of internal validation** |
| --- | --- | --- | --- | --- |
| Su et al., 2023 | NR | The Second Affiliated Hospital of Fujian Medical University, China. | Expert consensus | 10-fold cross-validation |
| Zhang et al.,2024 | NR | Zhongnan Hospital, Wuhan, China. | Expert consensus | Hand-out validation |
| Zhou et al.,2024 | NR | Zhongnan Hospital, Wuhan, China. | Expert consensus | 5-fold cross-validation |
| Zhang et al,2020 | 148Male/14Female | Zhongshan Hospital, Fudan University, China. | Expert consensus | Hand-out validation |
| Zhai et al.,2024 | 225Male/175Female | Center I and Center II (no specific name). | Expert consensus | NR |
| Yoo et al.,2024 | 158Male/242Female | Seoul National University Dental Hospital, Korea. | Expert consensus | Hand-out validation |
| Xu et al.,2022 | NR | NR | Expert consensus | 10-fold cross-validation |
| Xie et al.,2023 | NR | Luodian Hospital, Baoshan District, Shanghai, China. | Expert consensus | 10-fold cross-validation |
| Wei et al.,2023 | 227Male/218Female | Peking Union Medical College, China. | Expert consensus | 10-fold cross-validation |
| Ganitidis et al.,2021 | NR | Attikon General University Hospital, Athens. | Expert consensus | 4-fold cross-validation |
| Shi et al.,2023 | NR | General Hospital of Northern Theater Command, China. | Expert consensus & GE Healthcare Advantage Workstation(AW4.4, USA) | 5-fold cross-validation |
| Gui et al.,2023 | 86Male/18Female | Tianjin Huanhu Hospital, China and Tianjin First Central Hospital, China. | Expert consensus | 5-fold cross-validation |
| ALI et al.,2024 | NR | Politecnico di Torino, Italy. | NR | 5-fold cross-validation |
| Amitay et al.,2023 | 280Male/220Female | The Poriya Medical Center, Israel. | Expert consensus | 7-fold cross-validation |
| Ayoub et al.,2023 | 166Male/189Female | Changsha Central Hospital and the Second Affiliated Hospital of Fujian Medical University, China. | Expert consensus | NR |
| Cilla et al.,2022 | 19Male/11Female | The Gemelli Molise Hospital, Campobasso, Italy. | NR | NR |
| Guang et al.,2020 | 166Male/39Female | 10 Chinese hospitals in different regions. | Expert consensus | 3-fold cross-validation |
| He et al.,2024 | 386Male/779Female | The Shanghai Eighth People’s Hospital and Xinhua Hospital affiliated with Dalian University, China. | Expert consensus | Hand-out validation |
| Latha et al.,2022 | NR | The Bharat Scans, Chennai and the SRM Medical College Hospital and Research Center, Kattankulathur, Chennai, India. | Expert consensus | NR |
| Ma et al.,2021 | NR | Zhongnan Hospital, Wuhan, China. | Expert consensus | 5-fold cross-validation |
| Pisu et al.,2024 | 122Male/41Female | Azienda Ospedaliero-Universitaria Cagliari, Italy. | NR | 10-fold cross-validation |
| Wang et al.,2024 | 157Male/36Female | The First Hospital of Shanxi Medical University, China. | Expert consensus | 5-fold cross-validation |
| Gago et al.,2022 | NR | Girona’s Heart Registry | NR | 10-fold cross-validation |
| Omarov et al.,2024 | 9587Male/9912Female | UK Biobank (UKB) participants | NR | 5-fold cross-validation |
| Wang et al.,2023 | NR | The Second Affiliated Hospital of Fujian Medical University and Medical Imaging Center, Shenzhen Hospital, Southern Medical University, China. | Expert consensus | Hand-out validation |
| Vinayahalingam et al.,2024 | 3728Male/2438Female | The Department of Oral and Maxillofacial Surgery of Charit'e Hospital in Berlin, Germany. | Expert consensus | 10-fold cross-validation |
| Singh et al.,2024 | 58Male/42Female(Cyprus); 48Male/32Female(UK);  NR(CCA Database) | The Cyprus Institute of Neurology and Genetics, Nicosia, Cyprus; Saint Mary's Hospital, Imperial College of Medicine, Science and Technology, UK; CCA Database. | NR | 5-fold cross-validation |
| Shan et al.,2023 | 63Male/11Female | Beijing Hospital, China. | Expert consensus | Hand-out validation |
| Li et al.,2024 | 38Male/45Female | NR | Expert consensus | Hand-out validation |
| Jain et al.,2021 | 147Male/43Female | NR | Expert consensus | 10-fold cross-validation |
| Molinari et al.,2018 | NR | The Gradenigo hospital at Torino, Italy | Expert consensus | 3-,5-,7-,and 10-fold cross-validation |
| Kats et al.,2019 | NR | The School of Dental Medicine, Tel Aviv University, Israel. | Expert consensus | 10-fold cross-validation |
| Chen et al.,2022 | 91Male/24Female | The Renmin Hospital of Wuhan University, China. | NR | Hand-out validation |
| Zhao et al.,2025 | 311Male/334Female | The Renmin Hospital of Wuhan University, China. | NR | 10-fold cross-validation |
| Hu et al.,2025 | NR | The Fifth Affiliated Hospital of Wenzhou Medical University (center 1); the Second Affiliated Hospital of Wenzhou Medical University (center 2); the Lishui People’s Hospital (center 3) | NR | Hand-out validation |
| Li et al.,2025 | NR | Center 1 (the Fifth Affiliated Hospital of Wenzhou Medical University); Center 2 (the Second Affiliated Hospital of Wenzhou Medical University) | Expert consensus | 10-fold cross-validation |
| Yu et al.,2025 | 137Male/22Female | A hospital in North China | ^18^F-fluorodeoxyglucose ([^18^F]FDG) PET | 5-fold cross-validation |
| Lipai G D et al.,2025 | 139Male/80Female | Xuanwu Hospital | NR | Hand-out validation |
| Kuwada C et al.,2025 | 198Male/382Female | Three medical centers, in Cyprus, in the United Kingdom (UK), and in Greece. | Expert consensus | Hand-out validation |
| Lao et al.,2025 | 97Male/10Female | Department of Oral and Maxillofacial Radiology, Aichi Gakuin University School of Dentistry, Japan | Pathologic examination | 10-fold cross-validation |
| Abbreviations: NR not reported | | | | |

**Table S4. Study design and basic demographics of included 40 studies for the meta-analysis**

| **First author, year** | **Participants** | | **Open access data** | **N** | **Mean or median age (SD; range)** |
| --- | --- | --- | --- | --- | --- |
|  | **Inclusion criteria** | **Exclusion criteria** |  |  |  |
| Su et al.,2023 | Two-dimensional ultrasound diagnostic criteria for vulnerable plaques are as follows:  (a) The overall shape of the plaque is irregular;  (b) The fibrous surface cap is thin or not smooth;  (c) The internal lipid core is low or low to anechoic, heterogeneity, and basal lines;  (d) The echo-like continuity is poor or inconsistent, or there is an ulcer on the surface of the plaque. | NR | NO | 87 | NR |
| Zhang et al.,2024 | NR | NR | NO | 844 | NR |
| Zhou et al.,2024 | NR | NR | NO | 844 | NR |
| Zhang et al.,2020 | Patients with carotid artery stenosis greater than 30% and were evaluated on the same day using carotid plaque MRI and head MRI. | (a) Evidence of cardiogenic stroke;  (b) Bilateral TIA/stroke, brainstem involve ment only or undetermined hemispheric involvement;  (c) Primary intracranial diseases;  (d) Radiotherapy-induced carotid stenosis;  (e) Contraindications to MRI (such as pacemaker or severe claustrophobia) or to gadolinium. | NO | 162 | 66.80±7.37 |
| Zhai et al.,2024 | NR | (a) Poor image quality;  (b) Post arterial stent;  (c) Post aneurysmal clipping;  (d) Postthrombectomy. | NO | 240(Train) | 66.6±10.4 |
| Zhai et al.,2024 | NR | (a) Poor image quality;  (b) Post arterial stent;  (c) Post aneurysmal clipping;  (d) Postthrombectomy. | NO | 60（Internal Validation） | 68±8.4 |
| Zhai et al.,2024 | NR | (a) Poor image quality;  (b) Post arterial stent;  (c) Post aneurysmal clipping;  (d) Postthrombectomy. | NO | 100（External Validation） | 67.5±9 |
| Yoo et al.,2024 | (a) The study required participants to have a CT scan performed within one year before or after the PR (Panoramic Radiography);  (b) The PR was considered abnormal if calcification in the carotid artery was evident on both the PR and CT image and as normal if no calcification in the carotid artery was observed on either the PR or CT image. | (a) Inadequate image quality for diagnostic purposes on either the PR or CT image;  (b) Significant distortion or obscuration of the carotid artery region in the PR;  (c) Extensive surgical procedures carried out near the area of interest. | NO | 400 | 71.01±7.96 |
| Xu et al.,2022 | NR | NR | stable/vulnerable plaque | 126 | NR |
| Xie et al.,2023 | NR | NR | NO | 216 | NR |
| Wei et al.,2023 | NR | (a) Individuals with stroke;  (b) Patients under 40 years of age or over 80 years of age. | normal/abnormal | 445 | 54.6±7.8 |
| Ganitidis et al.,2021 | NR | NR | symptomatic/asymptomatic | 53 | NR |
| Shi et al.,2023 | NR | (a) Insufficient clinical data;  (b) Negative findings on carotid CTA;  (c) Presence of cerebral hemorrhage, tumor, trauma, or previous brain surgery;  (d) Posterior circulation stroke;  (e) Suspected cardioembolic sources;  (f) Carotid artery dissection;  (g) Accompanied by intracranial vessel diseases, such as atherosclerotic stenosis, aneurysm, or moyamoya disease;  (h) Previous carotid stenting and endarterectomy;  (i) Insufficient image quality for plaque analysis and radionics extraction. | YES | 167 | 66.2 ± 7.7 |
| Gui et al.,2023 | (a) Patients had an acute ischemic stroke within the past 7 days, whose corresponding unilateral infarction was confined to a single carotid region as defined by diffusion-weighted imaging;  (b) Patients with symptom duration ≤24 h met the WHO definition of transient ischemic attack but had documented acute ischemic infarction;  (c) Carotid lumen stenosis >30%. | (a) Patients with ≥70% carotid stenosis;  (b) Cardiogenic stroke;  (c) Patients with bilateral infarcts or clinical signs due to bilateral carotid plaques;  (d) Other causes(such as MRI images missing some slice data). | YES | 104 | 64 |
| ALI et al.,2024 | At least one of the following symptoms:  (a) Amaurosis;  (b) Transient ischemic attack (TIA);  (c) Minor stroke;  (d) Transient aphasia. | NR | NO | 420 | NR |
| Amitay et al.,2023 | (a) ≥40 years old；  (b) Had a panoramic radiograph encompassing both jaws(upper and lower), the hyoid bone, and the fourth upper cervical spine vertebrae. | (a) Low-quality panoramic radiographs with trimmed corners and/or blurred and spread spinal;  (b) Treatment with coumadin (warfarin);  (c) Diagnosis of hypomagnesemia;  (d) Diagnosis of hypercalcemia due to malignancy. | YES | 500 | 67.5±13.3 |
| Ayoub et al.,2023 | (a) All included plaques had a thickness of ≥2mm and were located in the common carotid artery or bifurcation, with neovascularization within the plaque.  (b) The plaques were initially diagnosed by conventional ultrasound, followed by ultrasonography contrast and/or MRI examination.  (c) Complete clinical information was available for all patients, informed consent was obtained, and the patients had no history of cranial surgery. | (a) Plaques with inhomogeneous internal echogenicity but significant calcification with acoustic shadowing  (b) Patients with a history of major cardiopulmonary pathology, severe trauma, or serious infection, those who cannot tolerate the procedure due to conditions such as combined congestive heart failure and severe liver and kidney dysfunction;  (c) Patients with contraindications to imaging or those who are allergic to the contrast agent of MRI;  (d) Patients with occlusion of the affected internal carotid artery and concomitant psychiatric disease were also excluded from the study. | NO | 355 | NR |
| Cilla et al.,2022 | (a) Aged>18 and<75 years;  (b) Diabetic and nondiabetic;  (c) With indications to receive carotid endarterectomy(TEA) for extracranial high-grade (>70%) internal carotid artery stenosis. | Patients with high-risk heart disease with carotid artery revascularization by stent angioplasty or myocardium and carotid artery revascularization by aortic coronary artery bypass surgery and carotid TEA. | NO | 30 | 72.96 |
| Guang et al.,2020 | (a) Symptomatic carotid plaque was defined by the presence of symptoms associated with previous ischaemic events on the ipsilateral side within the preceding 6 months.  (b) Patients were classified as asymptomatic if they had not experienced any stroke or transitory ischaemic attack in the previous 6 months the presence of carotid plaques and a maximum plaque thickness ≥2.0mm on grey scale ultrasound. | (a) Non-atherosclerotic disease;  (b) Shadowing of more than 50% due to plaque calcification on grey scale ultrasound;  (c) Previous endarterectomy or intravascular stent at the site of the index carotid artery;  (d) Contraindications to CEUS, such as unstable angina, acute cardiac failure, acute endocarditis, known right-to-left shunts and known allergy for microbubble contrast agents, and physical or mental inability to participate in the study. | YES | 205 | 61.6±8.4 |
| He et al.,2024 | NR | (a) Longitudinal ultrasound images were unavailable;  (b) Insufficient quality. | NO | 2013 | Modeling for detecting carotid plaques(No plaques:57.15±10.98;Plaques:68.09±12.46);  Modeling for assessing plaque stability(Stable:67.26± 11.32;Unstable:68.09±12.46) |
| Latha et al.,2022 | NR | NR | NO | NR | NR |
| Ma et al.,2021 | NR | NR | YES | 925 | NR |
| Pisu et al.,2024 | NR | (a) Time interval between symptom onset and imaging >1 week;  (b) Doubt with other pathologies(such as hypoglycemia, migraine, or post-paroxysmal neurological dysfunction);  (c) Concomitant intracranial pathology(such as brain tumor, abscess, or encephalitis);  (d) Cardiac embolic source;  (e) Symptomatic status due to posterior circulation occlusion. | NO | 175 | 72 |
| Wang et al.,2024 | NR | (a) Complete occlusion of the extracranial or cranial carotid artery;  (b) History of head injury, carotid stenting or endarterectomy, and intracranial artery bypass graft surgery;  (c) The extracranial or cranial or endarterectomy intracranial artery bypass extracranial or vulnerable plaque Vasculitis, aortic atrial fibrillation peripheral vascular, carotid artery vulnerable plaque, atrial fibrillation, valvular disease, peripheral vascular disease, carotid web ,and carotid artery dissection;  (d) Artery vulnerable plaque occurred than ipsilateral stroke or TIA having occurred more than 2 weeks before the head-and-neck CTA. | YES | 193 | 63.7±9.87 |
| Gago et al.,2022 | NR | NR | YES | 2379 | NR |
| Omarov et al.,2024 | NR | NR | YES | 19499 | aged 47-83 years |
| Wang et al.,2023 | (a) Individuals who require clarification of the nature of carotid artery stenosis based on diagnostic techniques such as ultrasound, CTA, and MRA; (b) Individuals with plaque formation detected on the arterial wall by ultrasound or CTA regardless of clinical symptoms;  (c) Individuals with clinical symptoms such as TIA and cerebral infarction of unknown origin despite other imaging techniques not detecting significant carotid artery stenosis;  (d) All individuals underwent magnetic resonance imaging of the carotid artery. | (a) Individuals with a severe allergic constitution;  (b) Contraindications to MRI;  (c) Patients with comorbidities (such as heart, lung, liver, kidney diseases);  (d) Individuals with mental illness or experienced cerebral hemorrhage. | YES | 87 | NR |
| Vinayahalingam et al.,2024 | NR | blurred and incomplete PRs | NO | 370 | NR |
| Singh et al.,2024 | NR | NR | YES | 190 | 54/54/27.5±3.5 |
| Shan et al.,2023 | (a) Adult patients over 18 years old;  (b) A diagnosis of CAP on CTA and CEUS;  (c) Relevant CTA and CEUS examinations that were performed simultaneously, not exceeding 3 weeks. | (a) Cases without available clinical records;  (b) CTA images of poor quality that could not extract radiomic features. | NO | 74 | 66.9±8.82 |
| Li et al.,2024 | Visible plaques identified by experts. | Degree of stenosis larger than 70%. | NO | 83 | NR |
| Jain et al.,2021 | NR | NR | NO | 190 | 68.78±10.88 |
| Molinari et al.,2018 | Subjects that showed at least one of the following symptoms:  (a) Amaurosis;  (b) Transient ischemic attack (TIA);  (c) Minor stroke;  (d) Transient aphasia;  (e) None of the subjects had a major stroke at the time of acquisition and none had other cerebrovascular diseases. | NR | NO | 2313 | 58.06±13.67 |
| Kats et al.,2019 | NR | NR | NO | 65 | NR |
| Chen et al.,2022 | (a) An acute ischemic stroke within the last 7 days in patients who had a corresponding unilateral infarct restricted to the territory of a single carotid artery defined by diffusion-weighted imaging;  (b) Patients with a symptom duration of ≤ 24h who had met the World Health Organization definition of transient ischemic attack but had a documented acute ischemic infarct;  (c) Carotid luminal stenosis >30%;  (d) Thickness of plaques confirmed to be larger than 2mm. | (a) Patients with carotid artery stenosis ≥70%;  (b) Cardiogenic stroke;  (c) Patients with bilateral infarct or clinical symptoms caused by bilateral carotid plaque;  (d) Other reasons(such as poor image quality of HRMRI). | NO | 115 | 51.2±13.8(Train);51.8 ± 12.2(Test) |
| Zhao et al.,2025 | (a)Based on the North American Symptomatic Carotid Endarterectomy Trial criteria[1], patients with a degree of carotid artery stenosis greater than 30% were included in the study;  (b)Having a carotid artery CTA and cranial magnetic resonance imaging (MRI) plain scan with a time interval of < 2 weeks;  (c)Patients with complete clinical information. | (a) A history of carotid endarterectomy or stent placement;  (b) Carotid dissection, aneurysm, primary intracranial disease, carotid stenosis caused by radiation therapy or vasculitis;  (c) Poor image quality;  (d) Complete occlusion of the carotid artery. | YES | 645 | Training set:69.41 ± 9.19(symptomatic) 70.66 ± 9.10(asymptomatic)  External validation set 1: 70.86 ± 9.71(symptomatic)，70.64 ± 9.25(asymptomatic) External validation set 2: 71.44 ± 12.37(symptomatic)，68.23 ± 8.92(asymptomatic) |
| Hu et al.,2025 | (a) Age > 18 years; (b) Carotid artery stenosis exceeding 30% as assessed by the North American Symptomatic Carotid Endarterectomy Trial (NASCET); (c) DECT CTA examination of the head and neck. | (a) Intracranial posterior circulation symptoms; (b) Carotid artery stenosis due to radiotherapy, vasculitis, or other causes; (c) Cardioembolic stroke; (d) History of carotid endarterectomy or stent implantation; (e) CTA indicates abnormal intracranial arterial lesions; (f) Presence of ulceration on the surface of the ascending aorta and/or significant aortic arch plaques that substantially affect the branches of the aortic arch; (g) Poor image quality. | YES | 416 | Training set: 73.13 ± 9.12(symptomatic)，71.99 ± 8.55(asymptomatic) Internal validation set: 72.70 ± 8.47(symptomatic)，70.60 ± 8.25(asymptomatic) External validation set: 72.86 ± 8.38 (symptomatic)，71.09 ± 9.13 (asymptomatic) |
| Li et al.,2025 | NR | (a) Incomplete data recorders; (b) Attendees younger than 18 years or older than 60 years; (c) Participants with cardiovascular disease. | YES | 2956 | Carotid plaque group:47.5±6.53 No carotid plaque group:38.6±7.73 |
| Yu et al.,2025 | (a) Aged 45–85 years; (b) Unilateral or bilateral carotid wall thickness >1.5 mm and visible plaque on ultrasonography according to the Mannheim consensus; (c) Ultrasound identified pathological stenosis with vulnerable plaque (e.g., irregular surface, ulcerations); (d) No contraindications for contrast-enhanced carotid wall MRI. | (a) Any ischemic cerebrovascular disease symptoms ipsilateral to the carotid stenosis 6 months prior; (b) Prior or scheduled carotid endarterectomy or carotid artery stent; (c) Any prior cancer or chemotherapy history; (d) Presence of acute or chronic inflammatory or autoimmune disease or use of chronic anti-inflammatory therapy at the time of PET/MRI; (e) Poor image quality; (f) Carotid wall volume <50 mm³ on MRI. | YES | 159 | 64.97 ± 7.76 |
| Lipai G D et al.,2025 | NR | NR | YES | 232 | NR |
| Kuwada C et al.,2025 | NR | NR | NO | 580 | 77 |
| Lao et al.,2025 | (a) Patients experienced transient ischemic attack or stroke, or other brain infarctions caused by plaque on the same side of the neck confirmed by ultrasound or MRI within the past 6 months; (b) Patients with facial or limb movement disorders caused by plaque on the same side of the neck; (c) Patients had to undergo ultrasound or MR imaging before undergoing CAS surgery, and the degree of stenosis caused by the plaque in the enrolled patients was ≥70%; (d) Patients underwent CTA imaging before CAS surgery, and the clinical data of enrolled patients were complete | The presence of old infarcts or hemorrhages. | NO | 107 | Training Group:65.8±3.8 Test Group：66.1±3.7 |
| Abbreviations: CEUS, contrast-enhanced ultrasound; CTA, computed tomography angiography; HRMRI, high resolution magnetic resonance imaging; MRI, magnetic resonance imaging; NR, not reported; TIA, transient ischemic attack. | | | | | |
|  | | | | | |

**Table S5. The sensitivity-specificity-and heterogeneity of all subgroups for the meta-analysis**

| **Subgroups** | **Number** | **Sensitivity (95%CI)** | **Specificity (95%CI)** | **SROC (95%CI)** | **SE (I^2^) (95%CI)** | **SP (I^2^) (95%CI)** |
| --- | --- | --- | --- | --- | --- | --- |
| All algorithms | 34 studies with 34 tables | 0.88 (0.85-0.91) | 0.89 (0.85-0.92) | 0.95 (0.92-0.96) | 93.58 (92.14-95.02) | 91.38 (89.26-93.49) |
| DL models | 24 studies with 24 tables | 0.88 (0.84-0.92) | 0.91 (0.86-0.94) | 0.95 (0.93-0.97) | 93.70 (92.02-95.38) | 95.55 (94.48-96.62) |
| ML models based on radiomics algorithms | 10 studies with 10 tables | 0.89 (0.82-0.93) | 0.83 (0.76-0.88) | 0.92 (0.89-0.94) | 90.20 (85.46-94.93) | 78.92 (66.34-91.49) |
| Models based on PRs imaging | 5 studies with 5 tables | 0.91 (0.80-0.96) | 0.93 (0.84-0.97) | 0.97 (0.95-0.98) | 82.28 (67.53-97.03) | 79.16 (61.12-97.20) |
| Models based on ultrasound imaging | 16 studies with 16 tables | 0.89(0.84-0.93) | 0.90 (0.84-0.94) | 0.95 (0.93-0.97) | 96.92 (96.10-97.73) | 94.98 (93.44-96.53) |
| Models based on MRI imaging | 5 studies with 5 tables | 0.87 (0.78-0.92) | 0.87 (0.76-0.93) | 0.93 (0.91-0.95) | 71.57 (45.22-97.93) | 73.21 (48.67-97.74) |
| Models based on CTA imaging | 8 studies with 8 tables | 0.83 (0.76-0.88) | 0.83 (0.75-0.89) | 0.90 (0.87-0.92) | 56.80 (22.76-90.85) | 83.79 (73.62,93.95) |
| Models utilizing transfer learning | 10 studies with 10 tables | 0.92 (0.87-0.95) | 0.93 (0.88-0.96) | 0.97 (0.95-0.98) | 79.84 (67.96-91.72) | 74.85 (59.17-90.54) |
| Models without transfer learning | 24 studies with 24 tables | 0.86 (0.82-0.90) | 0.86 (0.81-0.90) | 0.93 (0.90-0.95) | 94.12 (92.58-95.65) | 87.35 (83.20-91.50) |
| Presence or absence of carotid plaques | 11 studies with 11 tables | 0.89 (0.81-0.94) | 0.91 (0.86-0.95) | 0.96 (0.94-0.97) | 94.08 (91.74-96.42) | 97.60 (96.88-98.31) |
| Stable or vulnerable carotid plaques | 12 studies with 12 tables | 0.90 (0.85-0.94) | 0.91 (0.85-0.95) | 0.96 (0.94-0.97) | 95.19 (93.49-96.89) | 91.29 (87.61-94.97) |
| Symptomatic or asymptomatic carotid plaques | 10 studies with 10 tables | 0.86 (0.78-0.91) | 0.81 (0.74-0.87) | 0.90 (0.87-0.92) | 93.28 (90.37-96.18) | 84.67 (76.28-93.07) |
| Combined models | 7 studies with 7 tables | 0.85 (0.76-0.92) | 0.75 (0.70-0.80) | 0.77 (0.73-0.81) | 69.77 (46.02-93.53) | 40.08 (0.00-92.00) |
| Artificial intelligence models | 7 studies with 7 tables | 0.82 (0.74-0.88) | 0.74 (0.69-0.79) | 0.77 (0.73-0.80) | 62.97 (32.63-93.32) | 2.41 (0.00-100.00) |
| Testing | 27 studies with 27 tables | 0.90 (0.87-0.93) | 0.91 (0.87-0.93) | 0.96 (0.94-0.97) | 94.23 (92.82-95.64) | 93.45 (91.79-95.12) |
| External validation | 7 studies with 7 tables | 0.78 (0.71-0.83) | 0.80 (0.73-0.86) | 0.86 (0.82-0.88) | 66.67 (39.93-93.41) | 84.42 (74.01-94.82) |
| Sample size ≥200 | 14 studies with 14 tables | 0.91 (0.86,0.94) | 0.92 (0.87-0.95) | 0.97 (0.95-0.98) | 97.91 (97.38-98.43) | 97.40 (96.71-98.10) |
| Sample size <200 | 20 studies with 20 tables | 0.85 (0.80-0.88) | 0.86 (0.80-0.90) | 0.91 (0.89-0.94) | 60.64 (41.42-79.86) | 78.02 (68.78-87.26) |
| Multi-center studies | 9 studies with 9 tables | 0.84 (0.77-0.89) | 0.87 (0.81-0.91) | 0.92 (0.90-0.94) | 81.36 (69.99-92.74) | 80.24 (67.99-92.49) |
| Single-center studies | 22 studies with 22 tables | 0.89 (0.84-0.92) | 0.89 (0.84-0.93) | 0.95 (0.93-0.97) | 95.07 (93.80-96.35) | 90.63 (87.69-93.57) |
| Models based on ultrasound imaging | | | | | | |
| Presence or absence of carotid plaques | 5 studies with 5 tables | 0.88 (0.72-0.96) | 0.91 (0.80-0.96) | 0.95 (0.93-0.97) | 96.78 (95.13-98.43) | 97.97 (97.07-98.87) |
| Stable or vulnerable carotid plaques | 8 studies with 8 tables | 0.90 (0.84-0.94) | 0.92 (0.83-0.96) | 0.96 (0.94-0.97) | 97.01 (95.86-98.15) | 94.43 (91.85-97.00) |
| Different risk of bias studies | | | | | | |
| Low risk of bias studies | 5 studies with 5 tables | 0.80 (0.73-0.85) | 0.80 (0.71-0.87) | 0.86 (0.83-0.89) | 62.20 (25.32-99.07) | 87.10(77.19-97.00) |
| High/unclear risk of bias studies | 29 studies with 29 tables | 0.89 (0.86-0.92) | 0.90 (0.86-0.93) | 0.95 (0.93-0.97) | 94.61 (93.37-95.85) | 92.59 (90.71-94.48) |
| Abbreviations: SE-sensitivity; SP-specificity; DL-deep learning; ML-machine learning; CTA-computed tomography angiography; MRI-magnetic resonance imaging. | | | | | | |

**Table S6. Description of QUADAS-AI**

| **Domain** | **Signalling question** | **Concerns regarding “risk of bias”** |
| --- | --- | --- |
| **Subject selection** | Accurately characterize the source, size and quality of input data alongside clear patient eligibility criteria? | Risk of bias is judged as “low”, “high”, or “unclear”.  1. If all signalling questions for a domain are answered “yes” then risk of bias can be judged “low”.  2. If any signalling question is answered “no” this flags the potential for bias. Review authors then need to have in-depth discussions to judge risk of bias.  3. The “unclear” category should be used only when insufficient data are reported to permit a judgment. |
|  | Use non-open source data? |  |
|  | Present the rationale and breakdown of its training, validation and test sets? |  |
|  | Perform image pre-processing? |  |
|  | Provide the scanner model information used to acquire imaging data? |  |
| **Index test (AI)** | Performed adequate external evaluation ? |  |
| **Reference standard** | Was the reference standard likely to correctly classify the target condition? |  |
| **Work-flow** | Was the time between the index test and the reference standard reasonable? |  |

95% Confidence Contour: Reflects the uncertainty of the pooled estimate, being a range calculated from existing research data.

95% Prediction Contour: Reflects the range of results expected in new studies and focuses on the uncertainty of the pooled estimate from existing data, it accounts for potential variations in future new studies, with a usually wider range.

**Table S7. Sensitivity analysis model 1-3**

|  | Model 1 | Model 2 | Model 3 |
| --- | --- | --- | --- |
| t value | -1.42 | 0.19 | -1.34 |
| p value | 0.16 | 0.84 | 0.19 |

Model 1 is used to evaluate studies with excluded large sample sizes (sample size≥ 500) (n=7) compared to the original study. Model 2 is used to evaluate and exclude studies with small sample sizes (sample size≤50) (n=4) from the original study. Model 3 is used to evaluate and exclude studies (n=11) with extreme effect sizes (SE/SP>0.95 or<0.70) from the original study. Use linear regression to reflect the changes in diagnostic advantage ratios under different research combinations.

**Table S8. Diagnostic performance**

| **All studies** | **Sensitivity (95%CI)** | **Specificity (95%CI)** | **SROC (95%CI)** |
| --- | --- | --- | --- |
| All algorithms | 0.88 (0.85-0.91) | 0.89 (0.85-0.92) | 0.95 (0.92-0.96) |
| **Excluded study** | **Sensitivity (95%CI)** | **Specificity (95%CI)** | **SROC (95%CI)** |
| Model 1 | 0.86 (0.82-0.89) | 0.89 (0.84-0.92) | 0.93 (0.91-0.92) |
| Model 2 | 0.89 (0.85-0.92) | 0.89 (0.85-0.93) | 0.95 (0.93-0.96) |
| Model 3 | 0.86 (0.82-0.89) | 0.85 (0.82-0.89) | 0.92 (0.90-0.94) |
| Su et al., 2023 | 0.88 (0.85-0.91) | 0.89 (0.85-0.92) | 0.94 (0.92-0.96) |
| Zhou et al.,2024 | 0.89 (0.85-0.91) | 0.89 (0.85-0.92) | 0.95 (0.92-0.96) |
| Zhang et al.,2020 | 0.88 (0.85-0.91) | 0.89 (0.85-0.92) | 0.95 (0.92-0.96) |
| Zhai et al.,2024 | 0.88 (0.85-0.91) | 0.89 (0.85-0.92) | 0.95 (0.92-0.96) |
| Xu et al.,2022 | 0.88 (0.84-0.91) | 0.89 (0.85-0.92) | 0.94 (0.92-0.96) |
| Xie et al.,2023 | 0.88 (0.85-0.91) | 0.89 (0.85-0.92) | 0.95 (0.92-0.96) |
| Wei et al.,2023 | 0.87(0.84-0.90) | 0.88(0.84-0.91) | 0.94(0.92-0.96) |
| Ganitidis et al.,2021 | 0.88 (0.85-0.91) | 0.89 (0.85-0.92) | 0.95 (0.92-0.96) |
| Shi et al.,2023 | 0.89 (0.85-0.91) | 0.89 (0.85-0.92) | 0.95 (0.93-0.96) |
| Gui et al.,2023 | 0.89 (0.85-0.91) | 0.89 (0.85-0.92) | 0.95 (0.92-0.96) |
| Amitay et al.,2023 | 0.88(0.85-0.91) | 0.88(0.84-0.91) | 0.94(0.92-0.96) |
| Cilla et al.,2022 | 0.88 (0.85-0.91) | 0.89 (0.85-0.92) | 0.95 (0.92-0.96) |
| Guang et al.,2020 | 0.88 (0.85-0.91) | 0.89 (0.85-0.92) | 0.95 (0.92-0.96) |
| He et al.,2024 | 0.89 (0.85-0.91) | 0.89 (0.85-0.92) | 0.95 (0.92-0.96) |
| Ma et al.,2021 | 0.88(0.84-0.91) | 0.89(0.85-0.92) | 0.94(0.92-0.96) |
| Pisu et al.,2024 | 0.88 (0.85-0.91) | 0.89 (0.85-0.92) | 0.95 (0.92-0.96) |
| Gago et al.,2022 | 0.88(0.84-0.91) | 0.89(0.85-0.92) | 0.95(0.92-0.96) |
| Omarov et al.,2024 | 0.88 (0.85-0.91) | 0.89 (0.85-0.92) | 0.95 (0.92-0.96) |
| Wang et al.,2023 | 0.88 (0.84-0.91) | 0.89 (0.85-0.92) | 0.94 (0.92-0.96) |
| Vinayahalingam et al.,2024 | 0.88 (0.85-0.91) | 0.89 (0.85-0.92) | 0.95 (0.92-0.96) |
| Singh et al.,2024 | 0.88(0.84-0.91) | 0.88(0.84-0.91) | 0.94(0.92-0.96) |
| Shan et al.,2023 | 0.88 (0.85-0.91) | 0.89 (0.85-0.92) | 0.95 (0.92-0.96) |
| Li et al.,2024 | 0.89 (0.85-0.91) | 0.89 (0.85-0.92) | 0.95 (0.92-0.96) |
| Jain et al.,2021 | 0.88 (0.84-0.91) | 0.88 (0.84-0.91) | 0.94 (0.92-0.96) |
| Molinari et al.,2018 | 0.87(0.84-0.90) | 0.89(0.85-0.92) | 0.94(0.92-0.96) |
| Kats et al.,2019 | 0.88 (0.85-0.91) | 0.89 (0.85-0.92) | 0.95 (0.92-0.96) |
| Chen et al.,2022 | 0.88 (0.85-0.91) | 0.89 (0.85-0.92) | 0.95 (0.92-0.96) |
| Zhao et al.,2025 | 0.88(0.84-0.91) | 0.89(0.85-0.92) | 0.95(0.92-0.96) |
| Hu et al.,2025 | 0.88(0.85-0.91) | 0.89(0.85-0.92) | 0.95(0.92-0.96) |
| Yu et al.,2025 | 0.88(0.84-0.91) | 0.89(0.85-0.92) | 0.95(0.92-0.96) |
| Lipai G D et al.,2025 | 0.88(0.85-0.91) | 0.89(0.85-0.92) | 0.95(0.92-0.96) |
| Kuwada C et al.,2025 | 0.88(0.84-0.91) | 0.89(0.85-0.92) | 0.95(0.92-0.96) |
| Lao et al.,2025 | 0.88(0.84-0.91) | 0.89(0.85-0.92) | 0.95(0.92-0.96) |

After sequentially removing each study, no significant differences were observed in sensitivity (95%CI), specificity (95%CI), or SROC (95%CI).

**References**

36. Molinari F, Raghavendra U, Gudigar A, Meiburger KM, Rajendra Acharya U. An efficient data mining framework for the characterization of symptomatic and asymptomatic carotid plaque using bidimensional empirical mode decomposition technique. Med Biol Eng Comput. Sep 2018;56(9):1579-1593. [doi: 10.1007/s11517-018-1792-5] [Medline: 29473126]

37. Singh S, Jain PK, Sharma N, Pohit M, Roy S. Atherosclerotic plaque classification in carotid ultrasound images using machine learning and explainable deep learning. Intelligent Medicine. May 2024;4(2):83-95. [doi: 10.1016/j.imed.2023.05.003]

38. Li J, Huang Y, Song S, et al. Automatic diagnosis of carotid atherosclerosis using a portable freehand 3-D ultrasound imaging system. IEEE Trans Ultrason Ferroelectr Freq Control. Feb 2024;71(2):266-279. [doi: 10.1109/TUFFC.2023.3345740] [Medline: 38127609]

39. Yoo SW, Yang S, Kim JE, et al. CACSNet for automatic robust classification and segmentation of carotid artery calcification on panoramic radiographs using a cascaded deep learning network. Sci Rep. Jun 17, 2024;14(1):13894. [doi: 10.1038/s41598-024-64265-4] [Medline: 38886356]

40. Omarov M, Zhang L, Jorshery SD, et al. Automated deep learning-based detection of early atherosclerotic plaques in carotid ultrasound imaging. medRxiv. Sep 3, 2025:2024.10.17.24315675. [doi: 10.1101/2024.10.17.24315675] [Medline: 39484270]

41. Zhai D, Liu R, Liu Y, et al. Deep learning-based fully automatic screening of carotid artery plaques in computed tomography angiography: a multicenter study. Clin Radiol. Aug 2024;79(8):e994-e1002. [doi: 10.1016/j.crad.2024.04.015] [Medline: 38789330]

42. Vinayahalingam S, van Nistelrooij N, Xi T, et al. Detection of carotid plaques on panoramic radiographs using deep learning. J Dent. Dec 2024;151:105432. [doi: 10.1016/j.jdent.2024.105432] [Medline: 39461583]

43. Pisu F, Williamson BJ, Nardi V, et al. Machine learning detects symptomatic plaques in patients with carotid atherosclerosis on CT angiography. Circ Cardiovasc Imaging. Jun 2024;17(6):e016274. [doi: 10.1161/CIRCIMAGING.123.016274] [Medline: 38889214]

44. Zhou R, Gan W, Wang F, Yang Z, Huang Z, Gan H. Tri-correcting: label noise correction via triple CNN ensemble for carotid plaque ultrasound image classification. Biomed Signal Process Control. May 2024;91:105981. [doi: 10.1016/j.bspc.2024.105981]

45. Wang Y, Cai C, Du YM, et al. Assessment of stroke risk using MRI-VPD with automatic segmentation of carotid plaques and classification of plaque properties based on deep learning. J Radiat Res Appl Sci. Sep 2023;16(3):100630. [doi: 10.1016/j.jrras.2023.100630]

46. Shan D, Wang S, Wang J, et al. Computed tomography angiography-based radiomics model for predicting carotid atherosclerotic plaque vulnerability. Front Neurol. 2023;14:1151326. [doi: 10.3389/fneur.2023.1151326] [Medline: 37396779]

47. Xie J, Li Y, Xu X, et al. CPTV: classification by tracking of carotid plaque in ultrasound videos. Comput Med Imaging Graph. Mar 2023;104:102175. [doi: 10.1016/j.compmedimag.2022.102175] [Medline: 36630795]

48. Amitay M, Barnett-Itzhaki Z, Sudri S, et al. Deep convolution neural network for screening carotid calcification in dental panoramic radiographs. PLOS Digit Health. Apr 2023;2(4):e0000081. [doi: 10.1371/journal.pdig.0000081] [Medline: 37043433]

49. Gui C, Cao C, Zhang X, Zhang J, Ni G, Ming D. Radiomics and artificial neural networks modelling for identification of high-risk carotid plaques. Front Cardiovasc Med. 2023;10:1173769. [doi: 10.3389/fcvm.2023.1173769] [Medline: 37485276]

50. Shi J, Sun Y, Hou J, et al. Radiomics signatures of carotid plaque on computed tomography angiography: an approach to identify symptomatic plaques. Clin Neuroradiol. Dec 2023;33(4):931-941. [doi: 10.1007/s00062-023-01289-9] [Medline: 37195452]

51. Su SS, Li LY, Wang Y, Li YZ. Stroke risk prediction by color Doppler ultrasound of carotid artery-based deep learning using Inception V3 and VGG-16. Front Neurol. 2023;14:1111906. [doi: 10.3389/fneur.2023.1111906] [Medline: 36864909]

52. Chen S, Liu C, Chen X, Liu WV, Ma L, Zha Y. A radiomics approach to assess high risk carotid plaques: a non-invasive imaging biomarker, retrospective study. Front Neurol. 2022;13:35350403. [doi: 10.3389/fneur.2022.788652]

53. Gago L, Vila MDM, Grau M, Remeseiro B, Igual L. An end-to-end framework for intima media measurement and atherosclerotic plaque detection in the carotid artery. Comput Methods Programs Biomed. Aug 2022;223:106954. [doi: 10.1016/j.cmpb.2022.106954] [Medline: 35777216]

54. Jain PK, Sharma N, Saba L, et al. Automated deep learning-based paradigm for high-risk plaque detection in B-mode common carotid ultrasound scans: an asymptomatic Japanese cohort study. Int Angiol. Feb 2022;41(1):9-23. [doi: 10.23736/S0392-9590.21.04771-4] [Medline: 34825801]

55. Cilla S, Macchia G, Lenkowicz J, et al. CT angiography-based radiomics as a tool for carotid plaque characterization: a pilot study. Radiol Med. Jul 2022;127(7):743-753. [doi: 10.1007/s11547-022-01505-5] [Medline: 35680773]

56. Xu X, Huang L, Wu R, et al. Multi-feature fusion method for identifying carotid artery vulnerable plaque. IRBM. Aug 2022;43(4):272-278. [doi: 10.1016/j.irbm.2021.07.004]

57. Guang Y, He W, Ning B, et al. Deep learning-based carotid plaque vulnerability classification with multicentre contrast-enhanced ultrasound video: a comparative diagnostic study. BMJ Open. Aug 27, 2021;11(8):e047528. [doi: 10.1136/bmjopen-2020-047528] [Medline: 34452961]

58. Zhang R, Zhang Q, Ji A, et al. Identification of high-risk carotid plaque with MRI-based radiomics and machine learning. Eur Radiol. May 2021;31(5):3116-3126. [doi: 10.1007/s00330-020-07361-z] [Medline: 33068185]

59. Ma W, Cheng X, Xu X, et al. Multilevel strip pooling-based convolutional neural network for the classification of carotid plaque echogenicity. Comput Math Methods Med. 2021;2021:3425893. [doi: 10.1155/2021/3425893] [Medline: 34457035]

60. Ganitidis T, Athanasiou M, Dalakleidi K, Melanitis N, Golemati S, Nikita KS. Stratification of carotid atheromatous plaque using interpretable deep learning methods on B-mode ultrasound images. Annu Int Conf IEEE Eng Med Biol Soc. Nov 2021;2021:3902-3905. [doi: 10.1109/EMBC46164.2021.9630402] [Medline: 34892085]

61. Kats L, Vered M, Zlotogorski-Hurvitz A, Harpaz I. Atherosclerotic carotid plaque on panoramic radiographs: neural network detection. Int J Comput Dent. 2019;22(2):163-169. [Medline: 31134222]

62. Wei Y, Yang B, Wei L, et al. Real-time carotid plaque recognition from dynamic ultrasound videos based on artificial neural network. Ultraschall Med. Oct 2024;45(5):493-500. [doi: 10.1055/a-2180-8405] [Medline: 38113893]

63. Zhao T, Lin G, Chen W, et al. Predicting symptomatic carotid artery plaques with radiomics-based carotid perivascular adipose tissue characteristics: a multicenter, multiclassifier study. BMC Med Imaging. Aug 19, 2025;25(1):337. [doi: 10.1186/s12880-025-01876-x] [Medline: 40830841]

64. Hu W, Lin G, Chen W, et al. Radiomics based on dual-energy CT virtual monoenergetic images to identify symptomatic carotid plaques: a multicenter study. Sci Rep. Mar 26, 2025;15(1):10415. [doi: 10.1038/s41598-025-92855-3] [Medline: 40140428]

65. Liapi GD, Loizou CP, Griffin M, Pattichis CS, Nicolaides A, Kyriacou E. Transfer learning with class activation maps in compositions driving plaque classification in carotid ultrasound. Front Digit Health. 2025;7:1484231. [doi: 10.3389/fdgth.2025.1484231] [Medline: 40704367]

66. Yu F, Li X, Zhang Y, et al. MRI ensemble model of plaque and perivascular adipose tissue as PET-equivalent for identifying carotid atherosclerotic inflammation. EJNMMI Res. Aug 6, 2025;15(1):103. [doi: 10.1186/s13550-025-01293-9] [Medline: 40768105]

67. Kuwada C, Mitsuya Y, Fukuda M, et al. Area detection improves the person-based performance of a deep learning system for classifying the presence of carotid artery calcifications on panoramic radiographs. Oral Radiol. Jul 22, 2025;2025(1-10). [doi: 10.1007/s11282-025-00843-0] [Medline: 40694246]

68. Lao Q, Zhou R, Wu Y, et al. Predicting vulnerability status of carotid plaques using CTA-based quantitative analysis. J Cardiovasc Pharmacol. Mar 1, 2025;85(3):217-224. [doi: 10.1097/FJC.0000000000001664] [Medline: 39739382]

69. He L, Yang Z, Wang Y, et al. A deep learning algorithm to identify carotid plaques and assess their stability. Front Artif Intell. 2024;7:1321884. [doi: 10.3389/frai.2024.1321884] [Medline: 38952409]

70. Zhang Y, Gan H, Wang F, et al. A self-supervised fusion network for carotid plaque ultrasound image classification. Math Biosci Eng. Jan 31, 2024;21(2):3110-3128. [doi: 10.3934/mbe.2024138] [Medline: 38454721]

71. Ali T, Pathan S, Salvi M, Meiburger KM, Molinari F, Acharya UR. CAROTIDNet: a novel carotid symptomatic/asymptomatic plaque detection system using CNN-based tangent optimization algorithm in B-mode ultrasound images. IEEE Access. 2024;12:73970-73979. [doi: 10.1109/ACCESS.2024.3404023]

72. Ayoub M, Liao Z, Li L, Wong KKL. HViT: hybrid vision inspired transformer for the assessment of carotid artery plaque by addressing the cross-modality domain adaptation problem in MRI. Comput Med Imaging Graph. Oct 2023;109:102295. [doi: 10.1016/j.compmedimag.2023.102295] [Medline: 37717365]

73. Latha S, Muthu P, Lai KW, Khalil A, Dhanalakshmi S. Performance analysis of machine learning and deep learning architectures on early stroke detection using carotid artery ultrasound images. Front Aging Neurosci. 2021;13:828214. [doi: 10.3389/fnagi.2021.828214] [Medline: 35153728]

74. Wang L, Guo T, Wang L, et al. Improving radiomic modeling for the identification of symptomatic carotid atherosclerotic plaques using deep learning-based 3D super-resolution CT angiography. Heliyon. Apr 30, 2024;10(8):e29331. [doi: 10.1016/j.heliyon.2024.e29331] [Medline: 38644848]

75. Li YC, Zhang TR, Zhang F, et al. Development and validation of a carotid plaque risk prediction model for coal miners. Front Cardiovasc Med. 2025;12:1490961. [doi: 10.3389/fcvm.2025.1490961] [Medline: 40416817]
