# Supplementary figures and images for: Diagnostic Performance of Deep Learning and Radiomics in Extracranial Carotid Plaque Detection: Systematic Review and Meta-Analysis
Source: J Med Internet Res. 2026 Jan 22;28:e77092. doi: 10.2196/77092 (PMC12826653; doi:10.2196/77092)

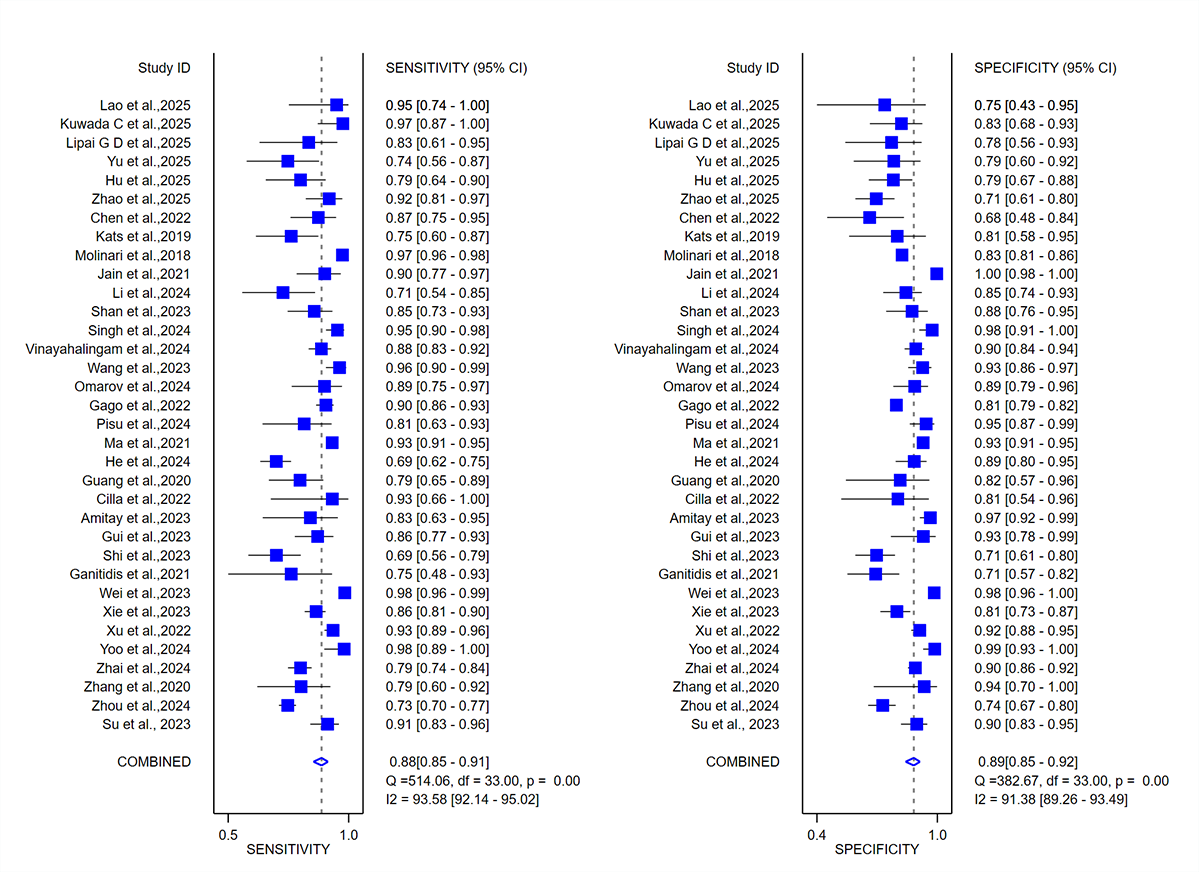

Supplement: Multimedia Appendix 2 [file jmir-v28-e77092-s002.png]

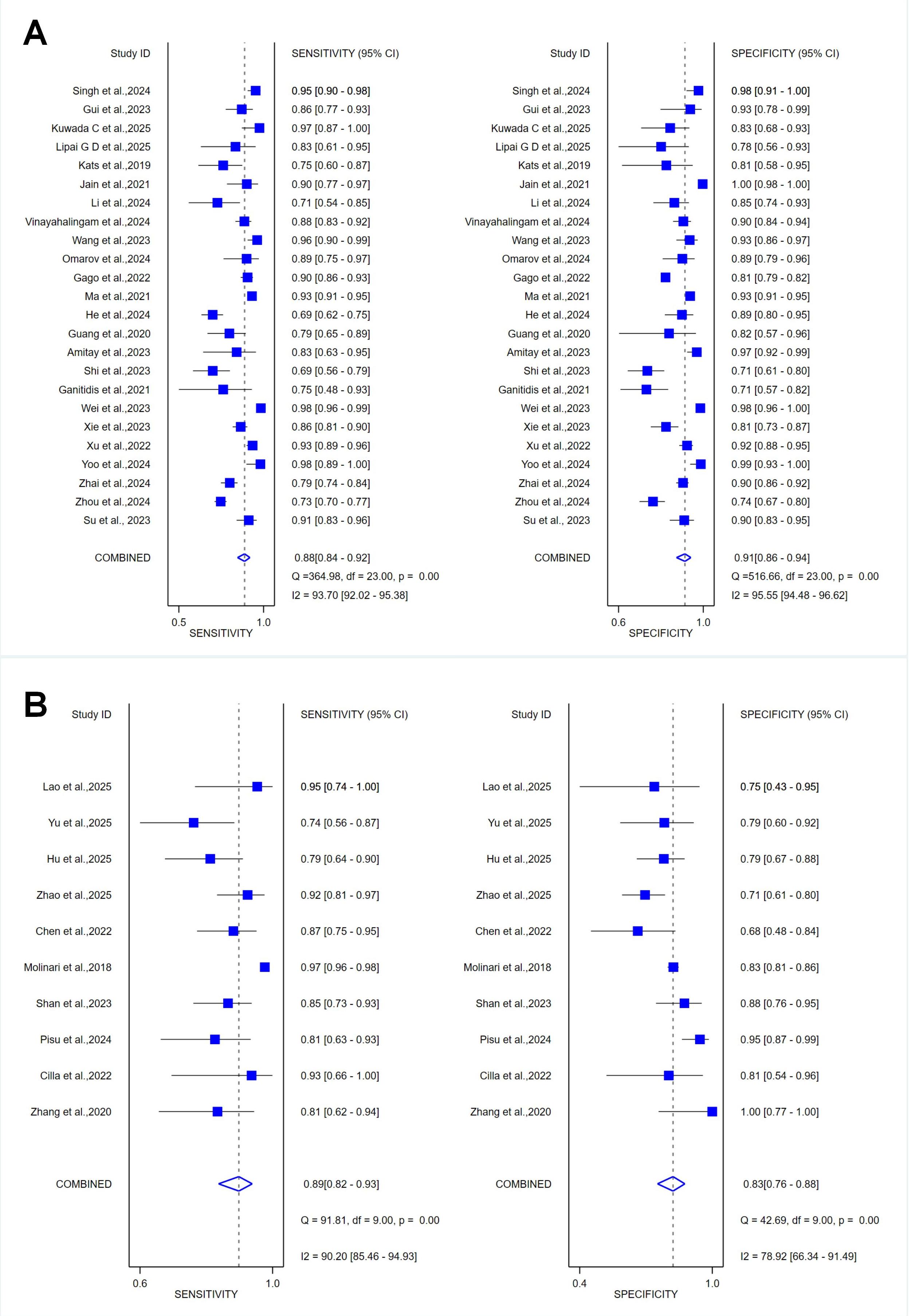

Supplement: Multimedia Appendix 3 [file jmir-v28-e77092-s003.jpg]

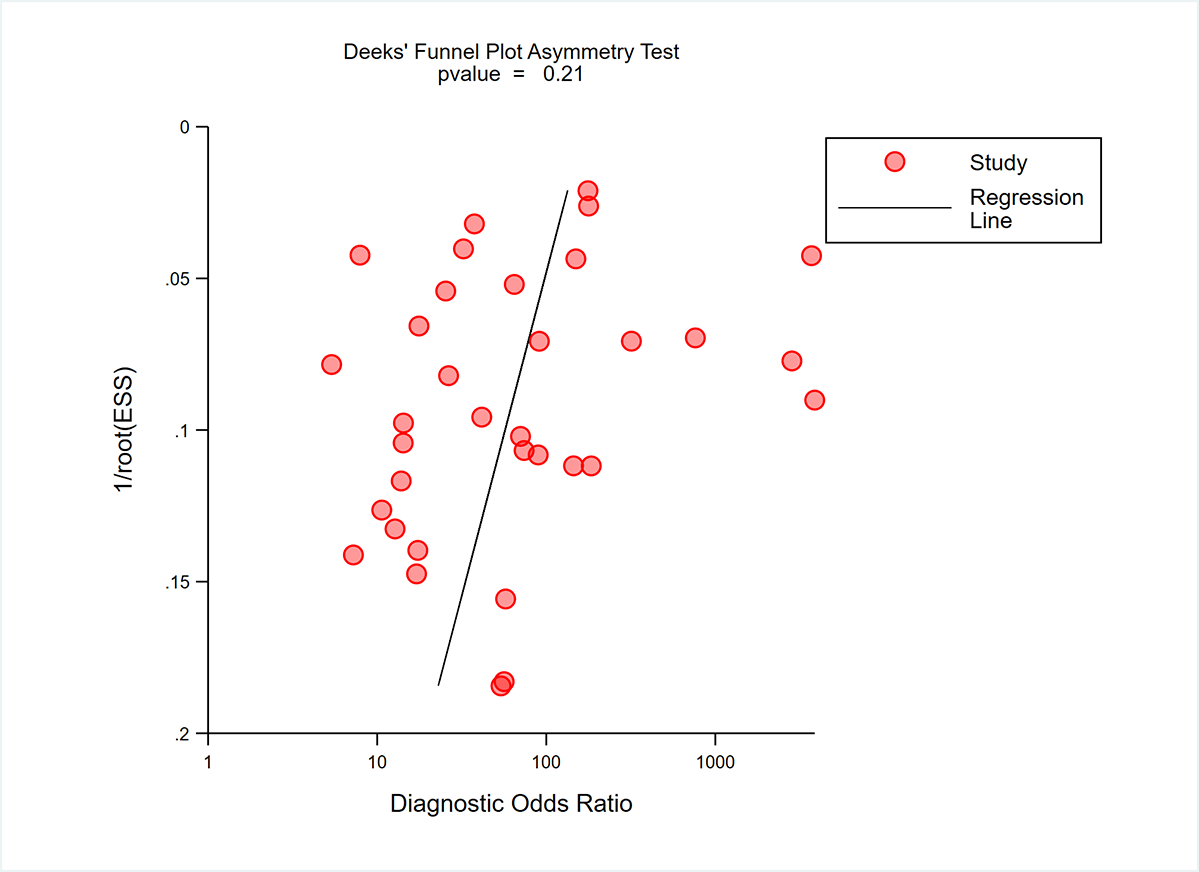

Supplement: Multimedia Appendix 4 [file jmir-v28-e77092-s004.png]

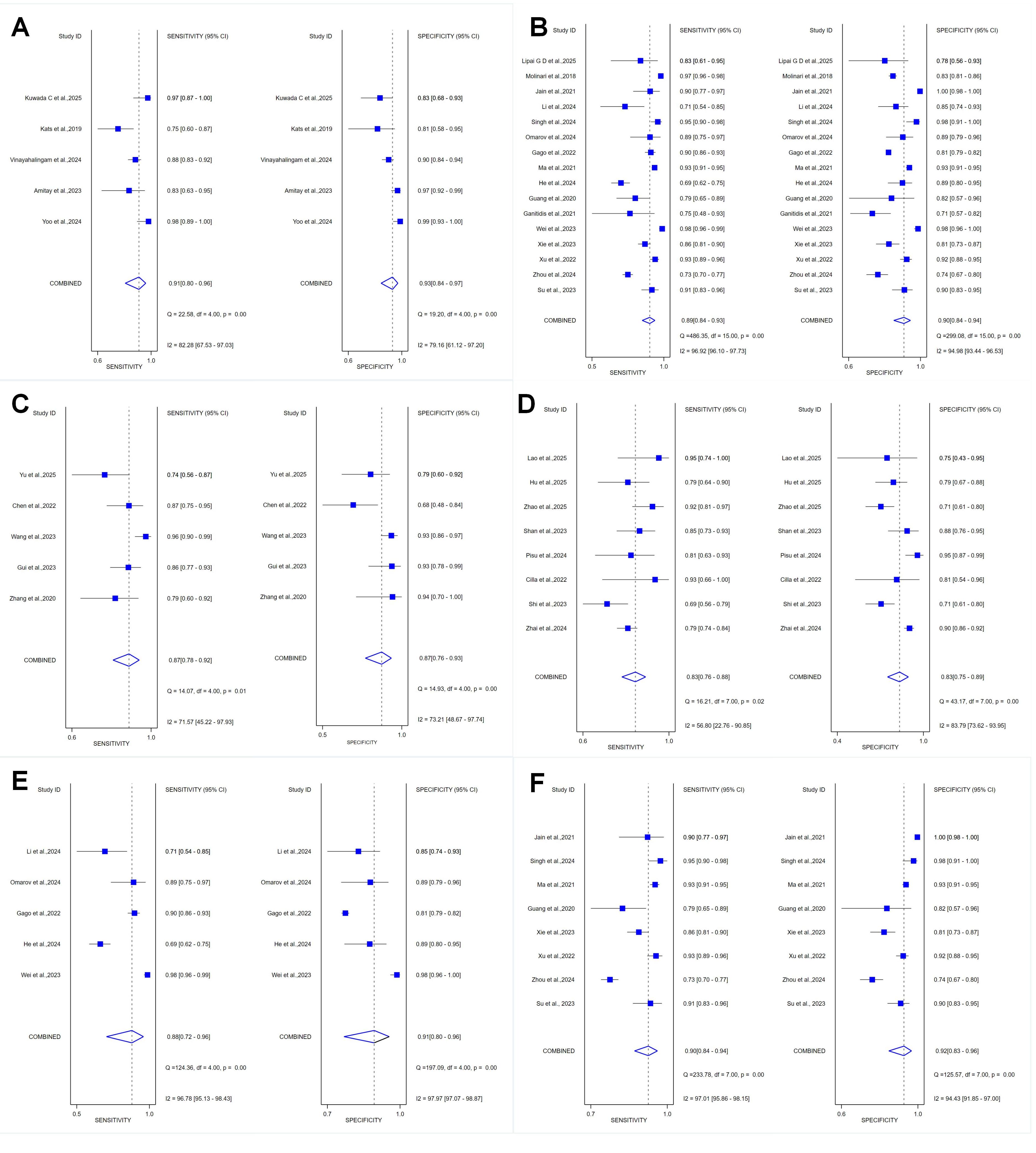

Supplement: Multimedia Appendix 5 [file jmir-v28-e77092-s005.jpg]

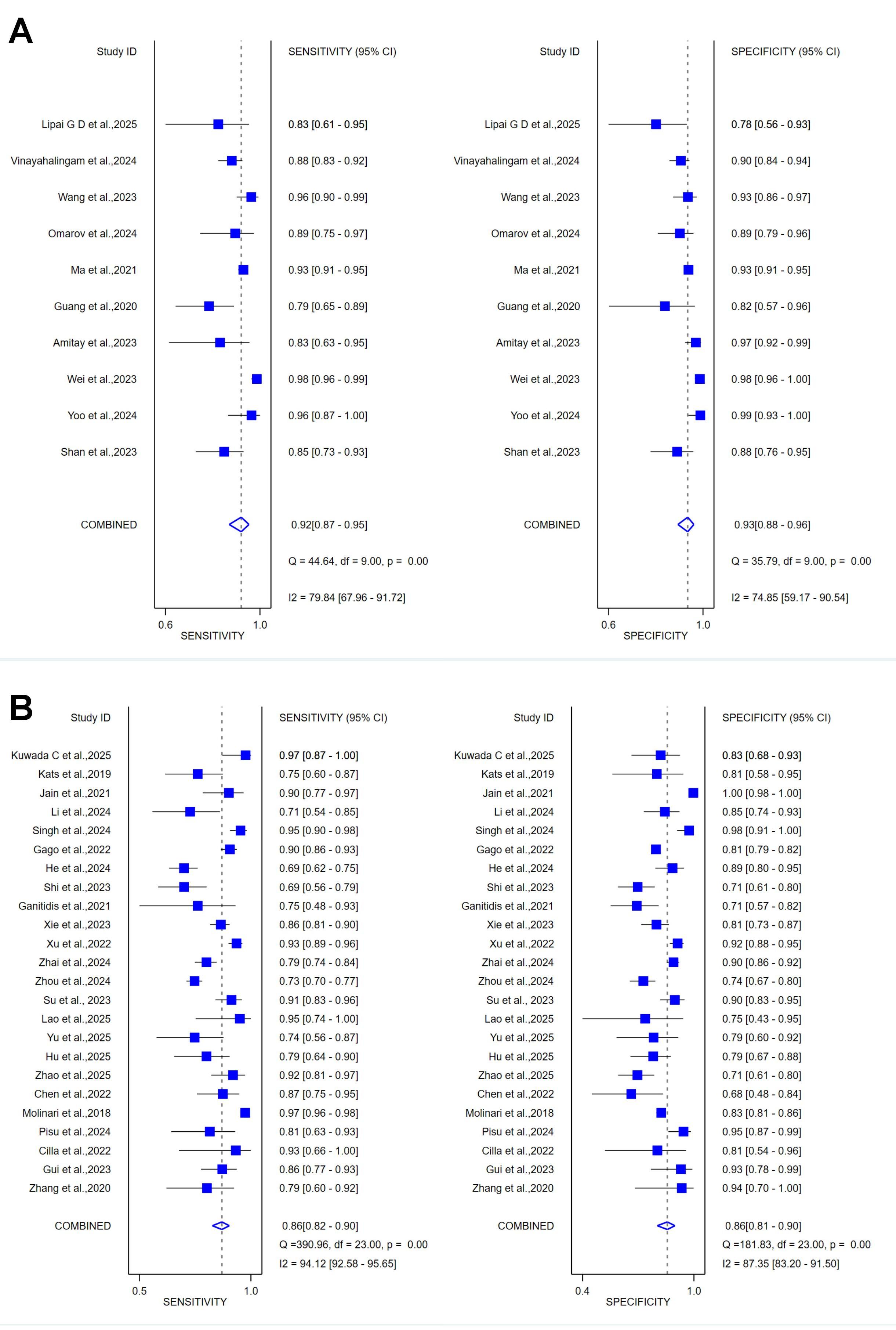

Supplement: Multimedia Appendix 6 [file jmir-v28-e77092-s006.jpg]

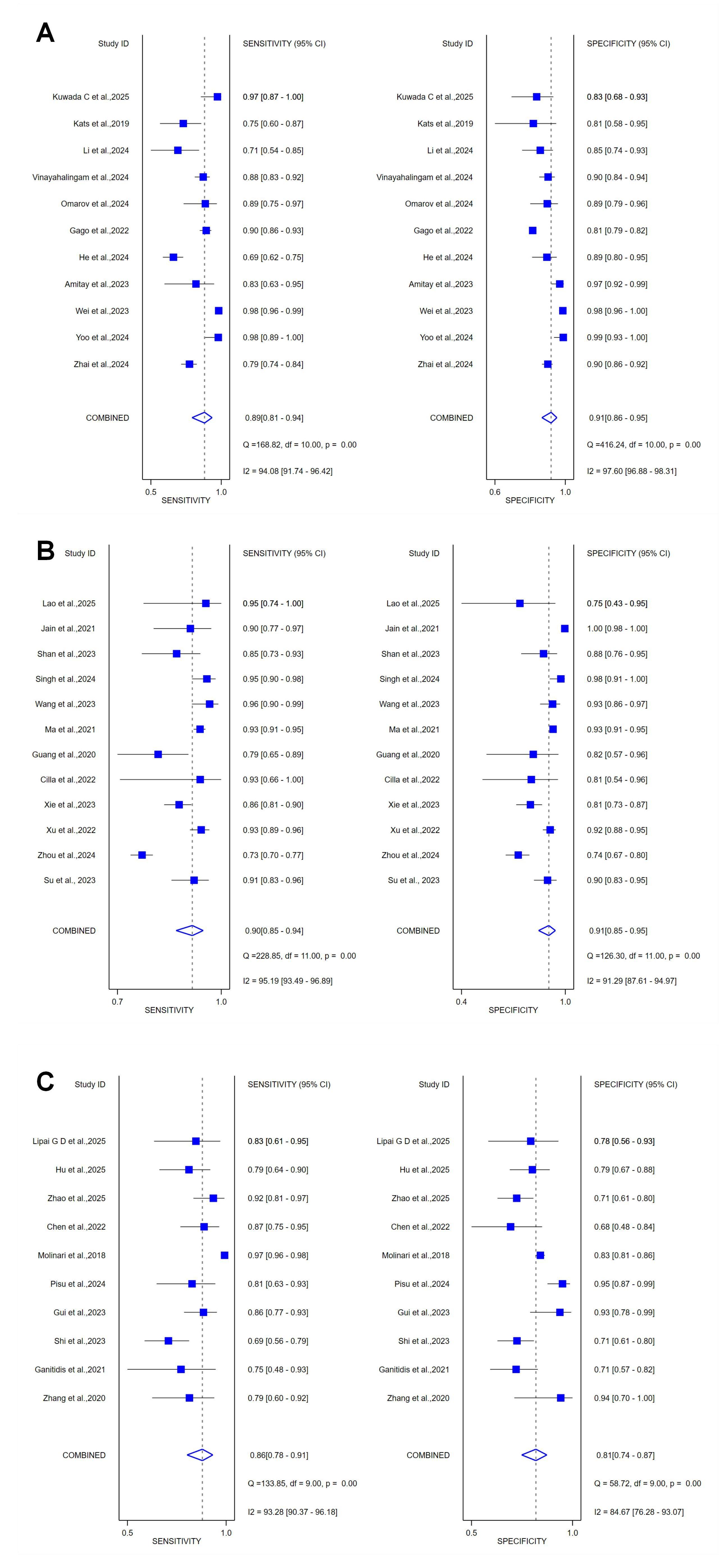

Supplement: Multimedia Appendix 7 [file jmir-v28-e77092-s007.jpg]

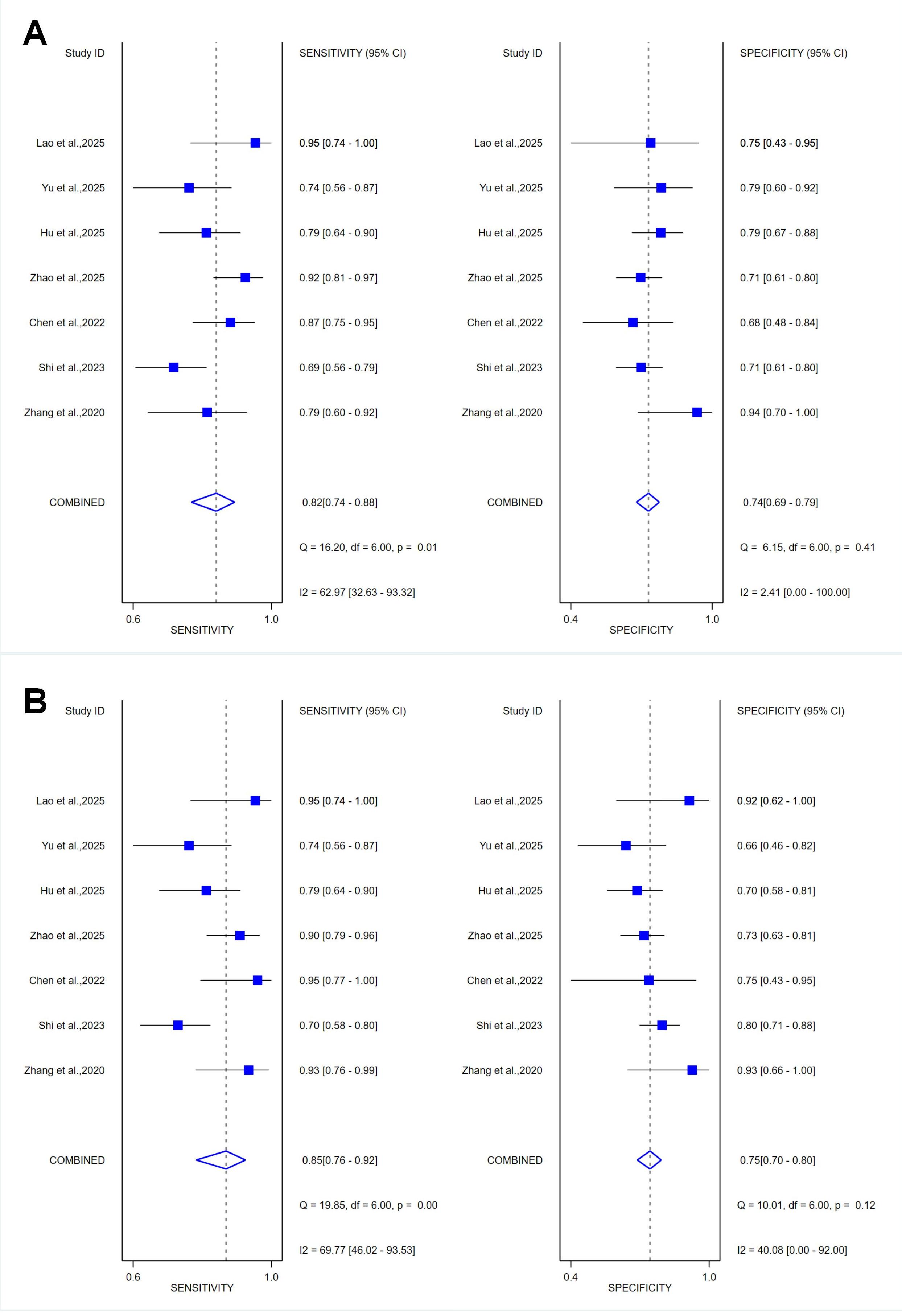

Supplement: Multimedia Appendix 8 [file jmir-v28-e77092-s008.jpg]

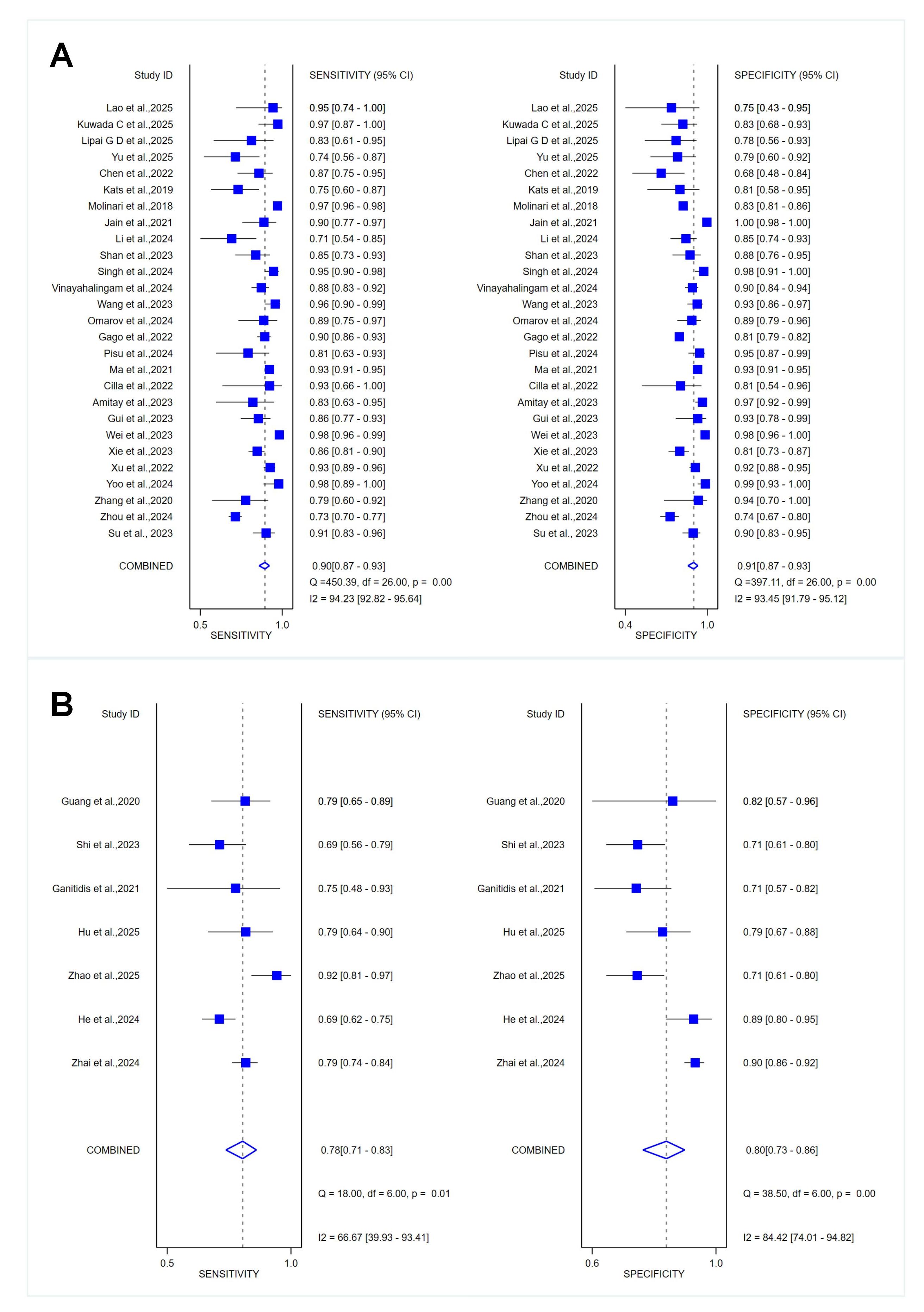

Supplement: Multimedia Appendix 9 [file jmir-v28-e77092-s009.jpg]

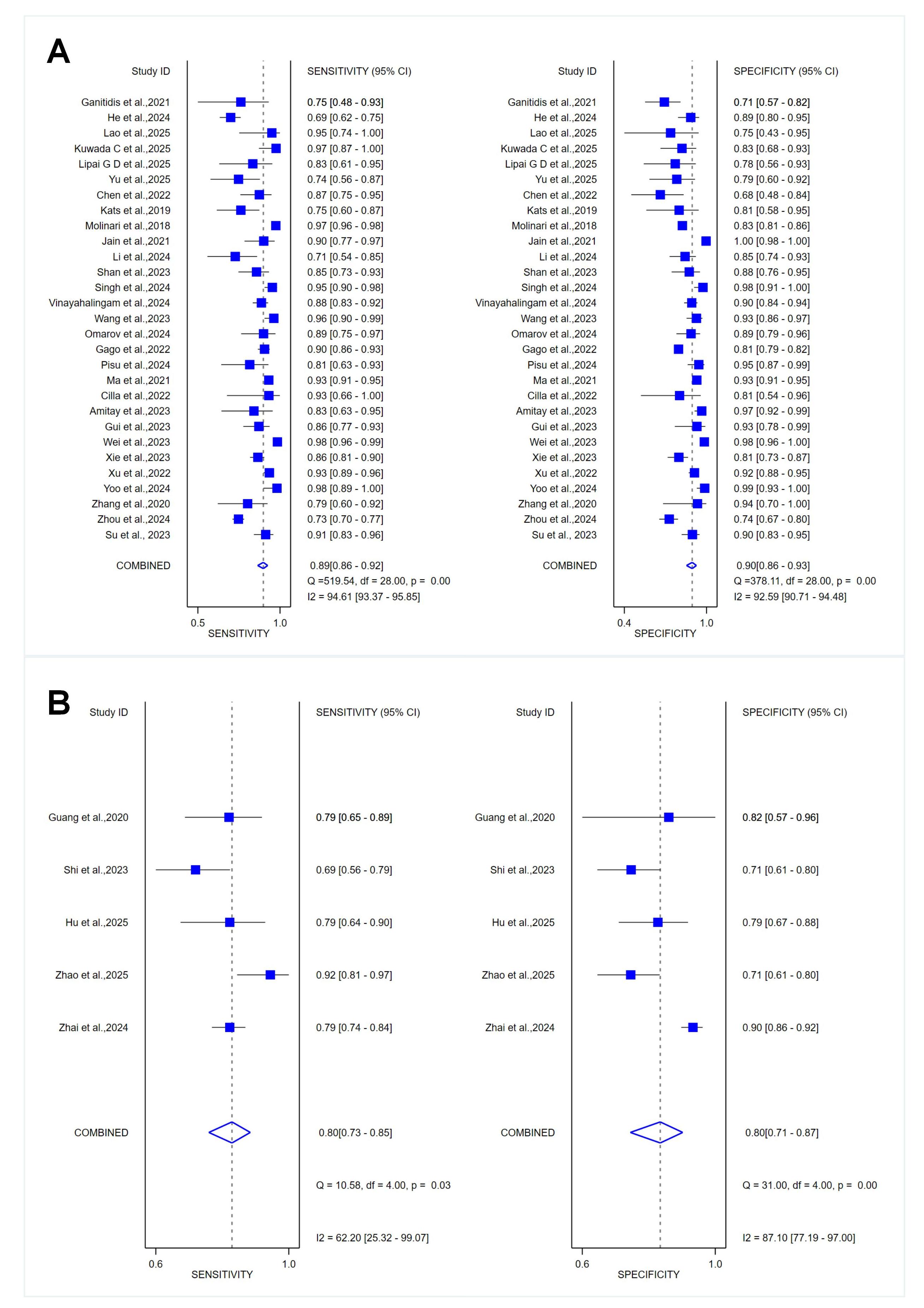

Supplement: Multimedia Appendix 10 [file jmir-v28-e77092-s010.jpg]

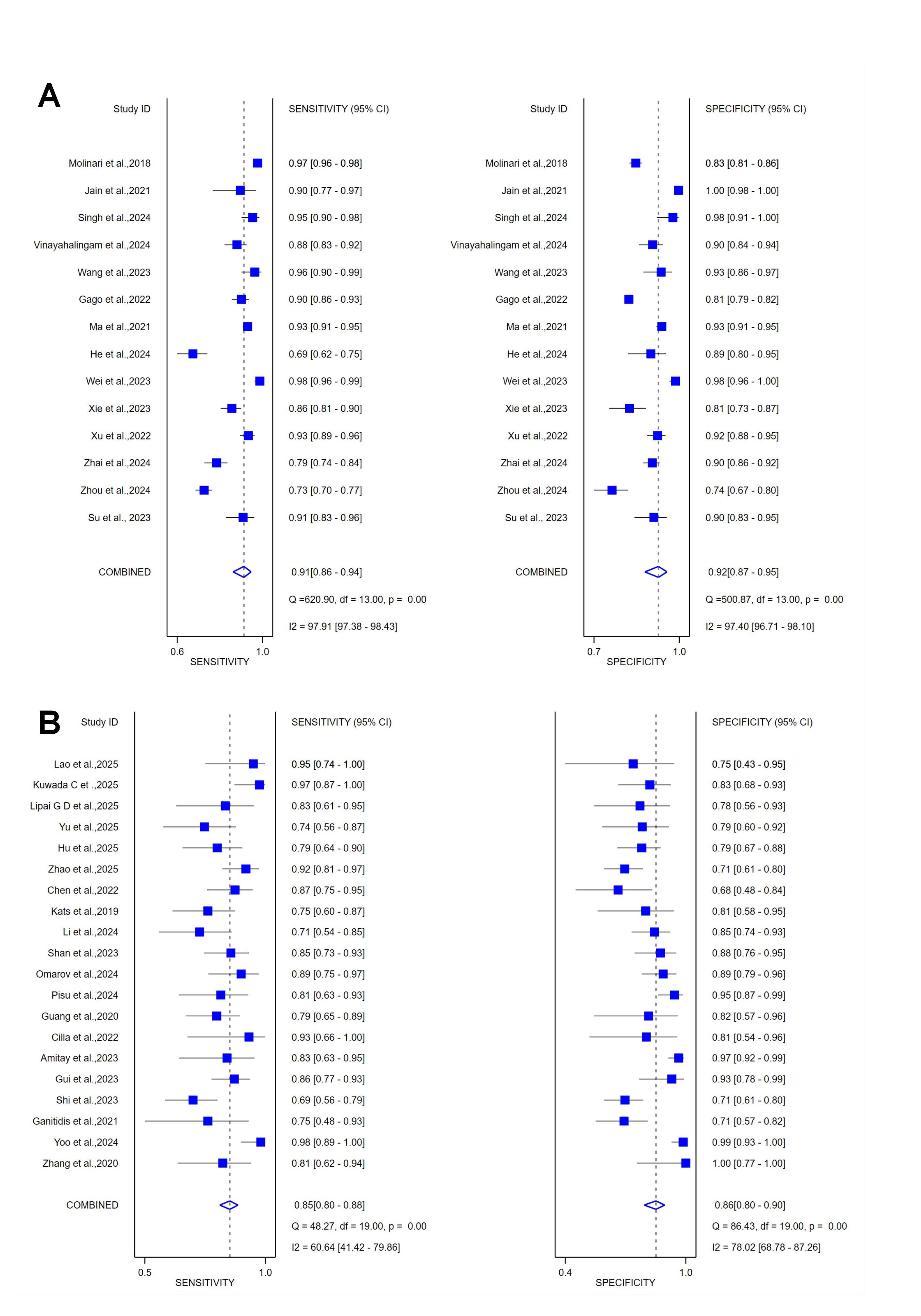

Supplement: Multimedia Appendix 11 [file jmir-v28-e77092-s011.jpg]

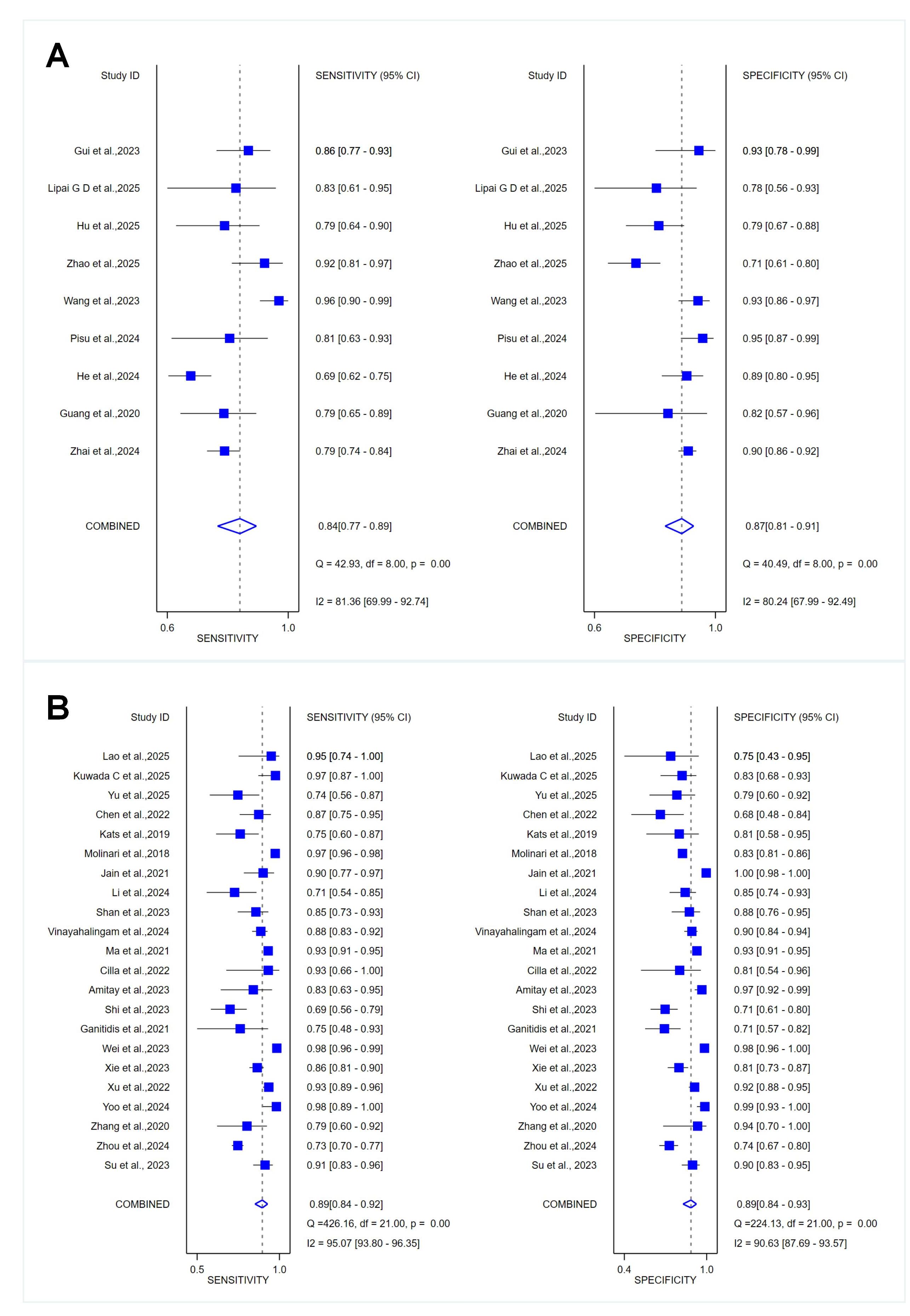

Supplement: Multimedia Appendix 12 [file jmir-v28-e77092-s012.jpg]

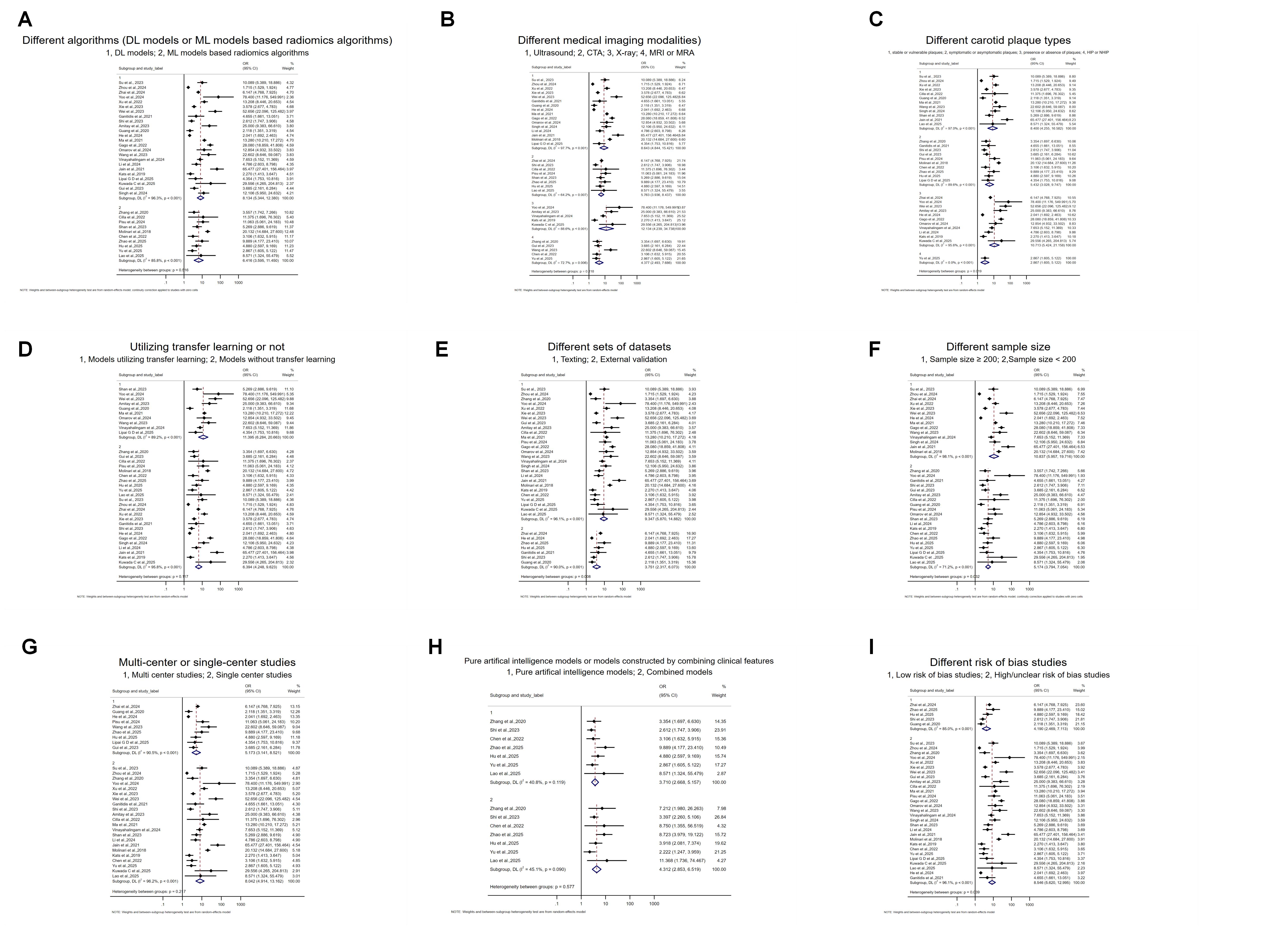

Supplement: Multimedia Appendix 13 [file jmir-v28-e77092-s013.jpg]

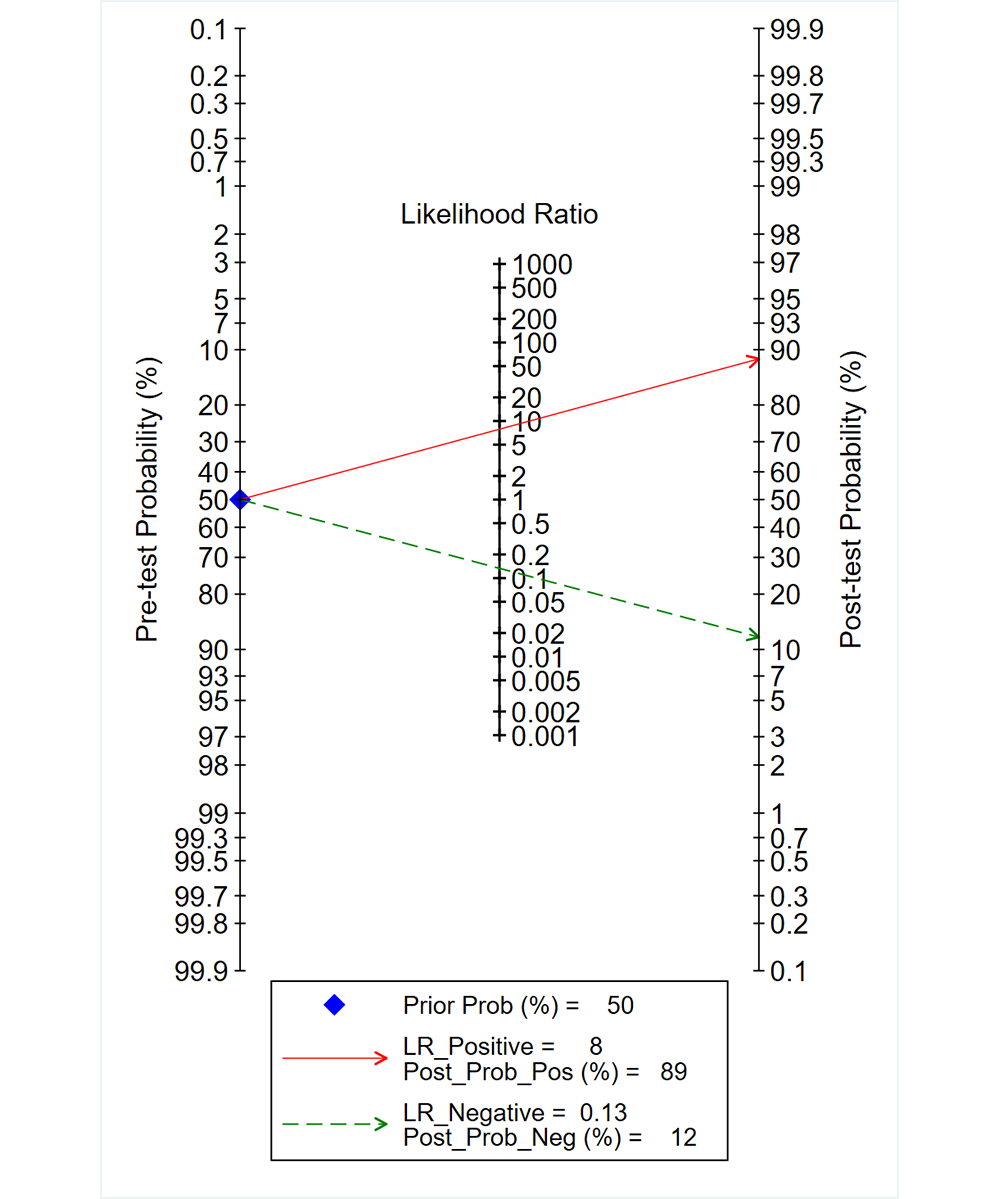

Supplement: Multimedia Appendix 14 [file jmir-v28-e77092-s014.png]

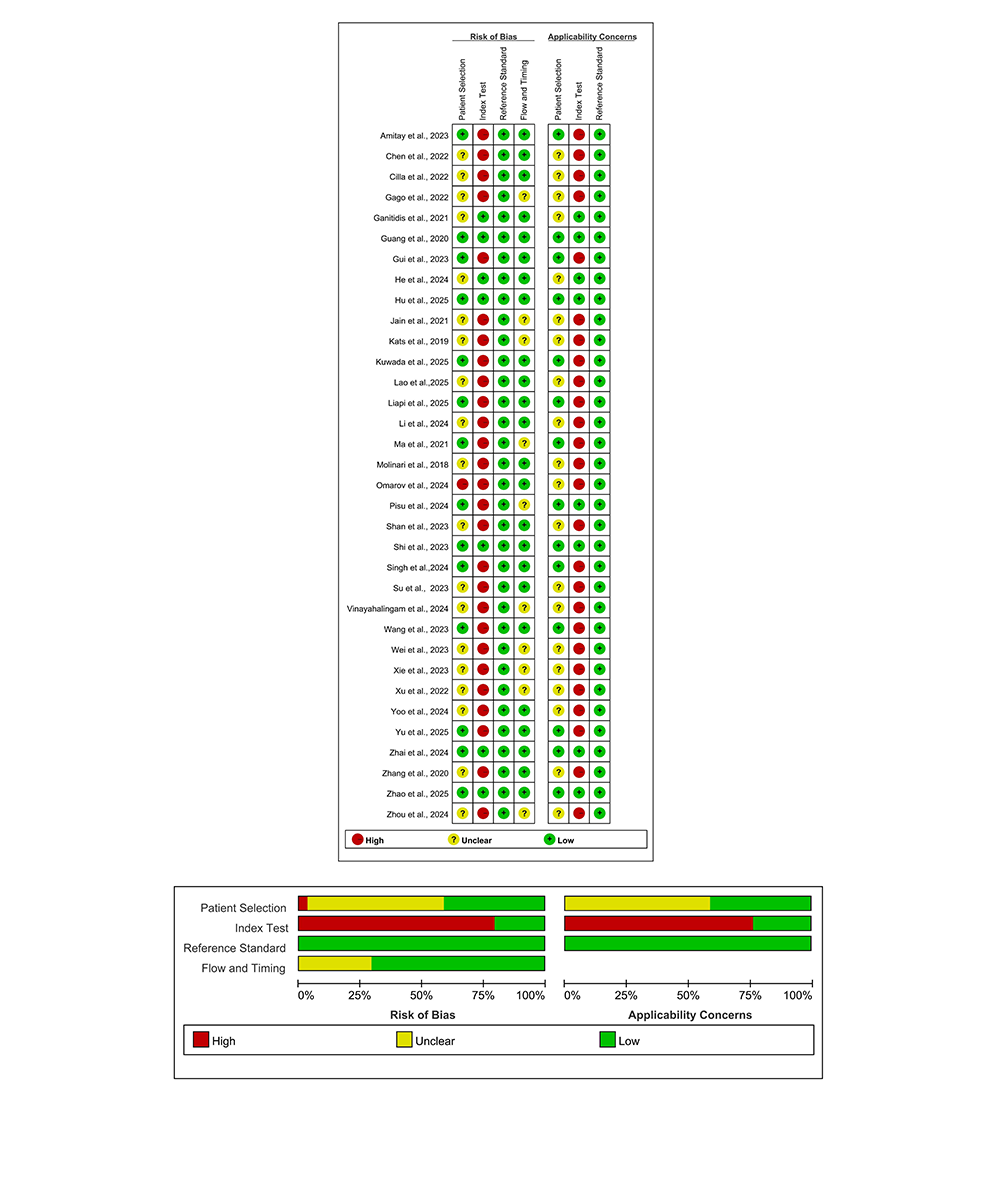

Supplement: Multimedia Appendix 15 [file jmir-v28-e77092-s015.png]
